# The NORDeHEALTH 2022 Patient Survey: Cross-Sectional Study of National Patient Portal Users in Norway, Sweden, Finland, and Estonia

**DOI:** 10.2196/47573

**Published:** 2023-11-13

**Authors:** Maria Hägglund, Anna Kharko, Josefin Hagström, Annika Bärkås, Charlotte Blease, Åsa Cajander, Catherine DesRoches, Asbjørn Johansen Fagerlund, Barbara Haage, Isto Huvila, Iiris Hörhammer, Bridget Kane, Gunnar O Klein, Eli Kristiansen, Kerli Luks, Jonas Moll, Irene Muli, Eline Hovstad Raphaug, Hanife Rexhepi, Sara Riggare, Peeter Ross, Isabella Scandurra, Saija Simola, Hedvig Soone, Bo Wang, Maedeh Ghorbanian Zolbin, Rose-Mharie Åhlfeldt, Sari Kujala, Monika Alise Johansen

**Affiliations:** 1 Participatory eHealth and Health Data Research Group Department of Women's and Children's Health Uppsala University Uppsala Sweden; 2 Medtech Science & Innovation Centre Uppsala University Hospital Uppsala Sweden; 3 School of Psychology Faculty of Health University of Plymouth Plymouth United Kingdom; 4 Department of Medicine, Division of General Medicine Beth Israel Deaconess Medical Center Harvard Medical School Boston, MA United States; 5 Department of Information Technology Uppsala University Uppsala Sweden; 6 Norwegian Centre for E-Health Research University Hospital of North Norway Tromsø Norway; 7 E-Medicine Centre Department of Health Technologies Tallinn University of Technology Tallinn Estonia; 8 Department of Archives Libraries & Museums Uppsala University Uppsala Sweden; 9 Department of Computer Science Aalto University Espoo Finland; 10 Business School Karlstad University Karlstad Sweden; 11 Centre for Empirical Research on Information Systems School of Business Örebro University Örebro Sweden; 12 School of Informatics University of Skövde Skövde Sweden; 13 Department of Clinical Medicine Telemedicine and E-health Research Group Arctic University of Norway Tromsø Norway

**Keywords:** electronic health record, patient-accessible electronic health record, online records access, health data, online medical record, patient access, patient portal, national survey

## Abstract

**Background:**

Although many surveys have been conducted on patients accessing their own health records in recent years, there is a limited amount of nationwide cross-country data available on patients’ views and preferences. To address this gap, an international survey of patient users was conducted in the Nordic eHealth project, NORDeHEALTH.

**Objective:**

We aimed to investigate the sociodemographic characteristics and experiences of patients who accessed their electronic health records (EHRs) through national patient portals in Norway, Sweden, Finland, and Estonia.

**Methods:**

A cross-sectional web-based survey was distributed using the national online health portals. The target participants were patients who accessed the national patient portals at the start of 2022 and who were aged ≥15 years. The survey included a mixture of close-ended and free-text questions about participant sociodemographics, usability experience, experiences with health care and the EHR, reasons for reading health records online, experience with errors, omissions and offense, opinions about security and privacy, and the usefulness of portal functions. In this paper, we summarized the data on participant demographics, past experience with health care, and the patient portal through descriptive statistics.

**Results:**

In total, 29,334 users completed the survey, of which 9503 (32.40%) were from Norway, 13,008 (44.35%) from Sweden, 4713 (16.07%) from Finland, and 2104 (7.17%) from Estonia. National samples were comparable according to reported gender, with about two-thirds identifying as women (19,904/29,302, 67.93%). Age distributions were similar across the countries, but Finland had older users while Estonia had younger users. The highest attained education and presence of health care education varied among the national samples. In all 4 countries, patients most commonly rated their health as “fair” (11,279/29,302, 38.48%). In Estonia, participants were more often inclined to rate their health positively, whereas Norway and Sweden had the highest proportion of negative health ratings. Across the whole sample, most patients received some care in the last 2 years (25,318/29,254, 86.55%). Mental health care was more common (6214/29,254, 21.24%) than oncological care (3664/29,254, 12.52%). Overall, most patients had accessed their health record “2 to 9 times” (11,546/29,306, 39.4%), with the most frequent users residing in Sweden, where about one-third of patients accessed it “more than 20 times” (4571/13,008, 35.14%).

**Conclusions:**

This is the first large-scale international survey to compare patient users’ sociodemographics and experiences with accessing their EHRs. Although the countries are in close geographic proximity and demonstrate similar advancements in giving their residents online records access, patient users in this survey differed. We will continue to investigate patients’ experiences and opinions about national patient-accessible EHRs through focused analyses of the national and combined data sets from the NORDeHEALTH 2022 Patient Survey.

## Introduction

### Background

The digitalization of health care is rapidly increasing, often with the aim of increasing patient safety, improving patient outcomes, and increasing efficiency [1]. Carefully implemented digital health care services have the potential to improve health care provision and strengthen opportunities for patient self-care, self-management, and shared decision-making [2,3]. Nordic countries are recognized as the forerunners of eHealth development and use [4]. This includes the use of online patient portals through which patients can gain online record access (ORA), often through services referred to as patient-accessible electronic health records (PAEHRs). Textbox 1 provides the definitions of key concepts relevant to patients’ ORA. This study focuses on electronic health records (EHRs) available through national patient portals in Nordic countries.

**Key terminology.**

**Electronic health record (EHR)**

- The WHO defines EHRs as “shared patient records that contain historical data about a patient that are compiled from all local Electronic Medical Records” [5]. In practice, the term EHR is however often used to describe both local electronic medical record systems and actual EHRs as defined by WHO. The ISO/TR 14292:2012(en) adds to the definition that EHRs are “healthcare provider-controlled” records [6], to distinguish them from personal health records.

**Personal health record (PHR)**

- The term PHR is used to describe health records that are controlled by the individual patient themselves, clearly distinguishing them from EHRs [6]. A PHR can be either tethered to an EHR (giving the patient access to information from the EHR), or untethered (stand-alone, requiring the patient to enter all health information themselves) [7], but is always controlled by the patient [6].

**Patient-accessible EHR (PAEHR)**

- As the term PHR has been used to describe both tethered and untethered solutions, and requiring the individual user to be in control of the information, the term PAEHR was proposed to describe a solution that gives patients online access to their EHR (but is often not controlled by the patient) [8].

**Online records access (ORA)**

- ORA has been used as a “solution-neutral” concept to describe the phenomenon of patients’ online record access [9]. ORA can be implemented through a tethered PHR, or a PAEHR, or any other technical solution that gives patients online record access.

**Open notes**

- Similar to the technology-neutral concept ORA, “open notes” has been used to describe the phenomenon of patients’ online access to free-text notes written by clinicians in the EHR [10]. Therefore, we consider open notes a key part of ORA, but ORA also includes other information, for example, laboratory results.

**Patient portal**

- Patient portals are online portals that are either locally provided by a specific health care provider or national patient portals as is the case in the Nordic countries. Patient portals were originally mostly for administration (appointment scheduling, secure messaging, prescription renewals, etc); however, they are now increasingly used to provide patients with ORA. In some patient portals, a PAEHR is provided as a specific service [11], whereas others may have more seamlessly integrated ORA through different patient portal functions. In a local patient portal, patients often have ORA from only 1 specific EHR system, whereas national patient portals can provide ORA to several EHR systems.

Although patients’ access to their EHRs is becoming the norm in Nordic countries, some challenges persist. In mental health care, for example, health care professionals (HCPs) may feel reluctant toward patient ORA, concerned that the contents may distress them [12-15]. The management of a dependent’s EHR through proxy access is not possible for some patient groups [16]. Adoption and actual use among patients also vary, with some struggling to use the digital services enabling ORA. Furthermore, some users may be at risk of experiencing negative consequences from ORA [17]. Thus, the ongoing study of the current implementation of ORA is not only necessary but also timely on a global scale.

In the European Union (EU), patients will soon gain greater control over their EHR, including cross-border access within the EU to their electronic health data through the new European Health Data Space initiative [18]. In the United States, since 2021, the federal rule from the 21st Century Cures Act mandates US health care providers to offer patients access to all health information in their EHRs without charge [19]. In the United Kingdom, patient ORA is gradually being implemented through the NHS Long Term Plan, which promises full access by 2024 [20]. Considering the global trend for the implementation of ORA, gaining an in-depth perspective of how Nordic patients view and use their national EHRs is of international importance.

### The National Health Care Systems and Patient Portals

Norway has universal health and social insurance coverage that is funded by general taxes, and patients pay a low fee or copayment for most services. The Norwegian health care system is organized into 2 levels: the state is responsible for specialist services and the municipalities are responsible for primary care. The EHR has been fully established for many years, and the patient is both the subject and the owner of the health record. Since 2001, patients have had the legal right to access their health records [21], and in 2013, a white paper stated that patients should have digital access [22]. In 2022, 3 of 4 health regions offered patients aged ≥16 years and parents of children aged ≤12 years digital access to their hospital’s EHR via the National Health Portal *Helsenorge.no*. In general, all documents available in digital format, including psychiatry reports, are made available for patients as soon as they are signed off by HCPs, unless the HCPs decide to deny access. At the beginning of 2022, Norway had approximately 5.4 million residents, and there were 13.3 million visits and 8.1 million log-ins to Helsenorge.no in February [23].

Swedish health care is similar to that of Norway in that it upholds the principles of universal coverage and publicly funded health care, ensuring access to comprehensive services for all residents [24]. In Sweden, the health care system is decentralized, meaning that it is the responsibility of regions and municipalities to overlook medical care provision while being guided by the central government. In 2008, the government stipulated that all patients should have a single online access point to health care, which was implemented through the national patient portal *1177.se.* Using government-approved electronic ID authentication, users gain access to a variety of personalized digital health care services, for example, they can find health care providers, book appointments, and send secure messages. To view their EHR, including the list of prescriptions, test results, and consultation notes from primary and secondary care, patients use the PAEHR service *Journalen* available on the 1177 portal. *Journalen* was first implemented in the Uppsala region in 2012 and later migrated to the 1177 national patient portal [11] and made available in all regions. Since the start of the pandemic, the national portal has dramatically increased in popularity with more users visiting, primarily through a mobile device [11]. The government agency Statistics Sweden reported that Sweden had approximately 10.5 million residents in 2022 [25]. In January 2023, 8.1 million log-ins to the portal were made by 2.8 million unique users [26].

The Finnish health care system is similarly based on public health care services to which everyone residing in the country is entitled. According to the Constitution of Finland, the public authorities should guarantee for everyone adequate social, health, and medical services [27]. Until 2023, health care in Finland consisted of a highly decentralized 3-level publicly funded health care system and a much smaller private sector. As of the social and health care reform of 2023, there are now 21 new well-being service counties in Finland and the City of Helsinki responsible for organizing health, social, and rescue services for citizens in the area. The Finnish national patient portal *My Kanta* was first introduced in 2010, and different functions were adopted in a step-by-step manner [28]. Since 2015, the My Kanta patient portal has provided all Finnish citizens who use public health care with access to their health records and prescriptions, as well as the possibility of renewing the latter [28]. Patients have access to both their primary and secondary care records. The population of Finland was 5.6 million in 2021 and, in the same year, 92% of adults aged between 18 and 65 years used the patient portal [29].

In Estonia, access to free health care is ensured through the Estonian Health Insurance Fund, compulsory solidarity-based health insurance paid by employers or subsidized by the government. Health care providers are managed at a municipal, governmental, or private level. General practitioners, for example, are private entrepreneurs, but most hospitals are publicly owned. Municipalities can oversee the organization of health care but to a lesser degree [30]. The nationwide health information system in Estonia, which includes the national patient portal *Digilugu.ee*, has been operating since the end of 2008 [31]. Since the beginning of 2009, all health care providers have the obligation to share a number of standardized medical documents with the PAEHR [32]. However, the different types of mandatory documents have increased with time and different functions have been added in a step-by-step manner when a new document or data have been digitized. Since 2010, all residents with electronic ID have been able to see their health records, laboratory and examination results, diagnosis, and prescriptions from primary and secondary care. In addition, in 2019 a nationwide appointment booking system was added [33]. The population of Estonia was 1.3 million in 2022 [34] and between January and March 2022, 0.6 million users logged into the national portal.

### The NORDeHEALTH 2022 Patient Survey

Currently, most studies on patient ORA have collected data from 1 country or region [10,29,35,36], impeding international comparisons. However, some studies assessing international policies exist [37]. Furthermore, many studies have focused on only one part of the EHR, for example, open notes [10]. The NORDeHEALTH research project [38,39], with partners from Norway, Sweden, Finland, Estonia, and the United States, strives to evaluate the implementation of patient ORA through joint research. In 2022, we conducted a patient survey in Norway, Sweden, Finland, and Estonia to study patients’ opinions and experiences with the PAEHR provided through the national patient portals.

In this paper, we aim to describe the methodology for the NORDeHEALTH 2022 Patient Survey and present an overview of the data collected in the 4 countries.

## Methods

### Survey Structure

**Figure 1:**
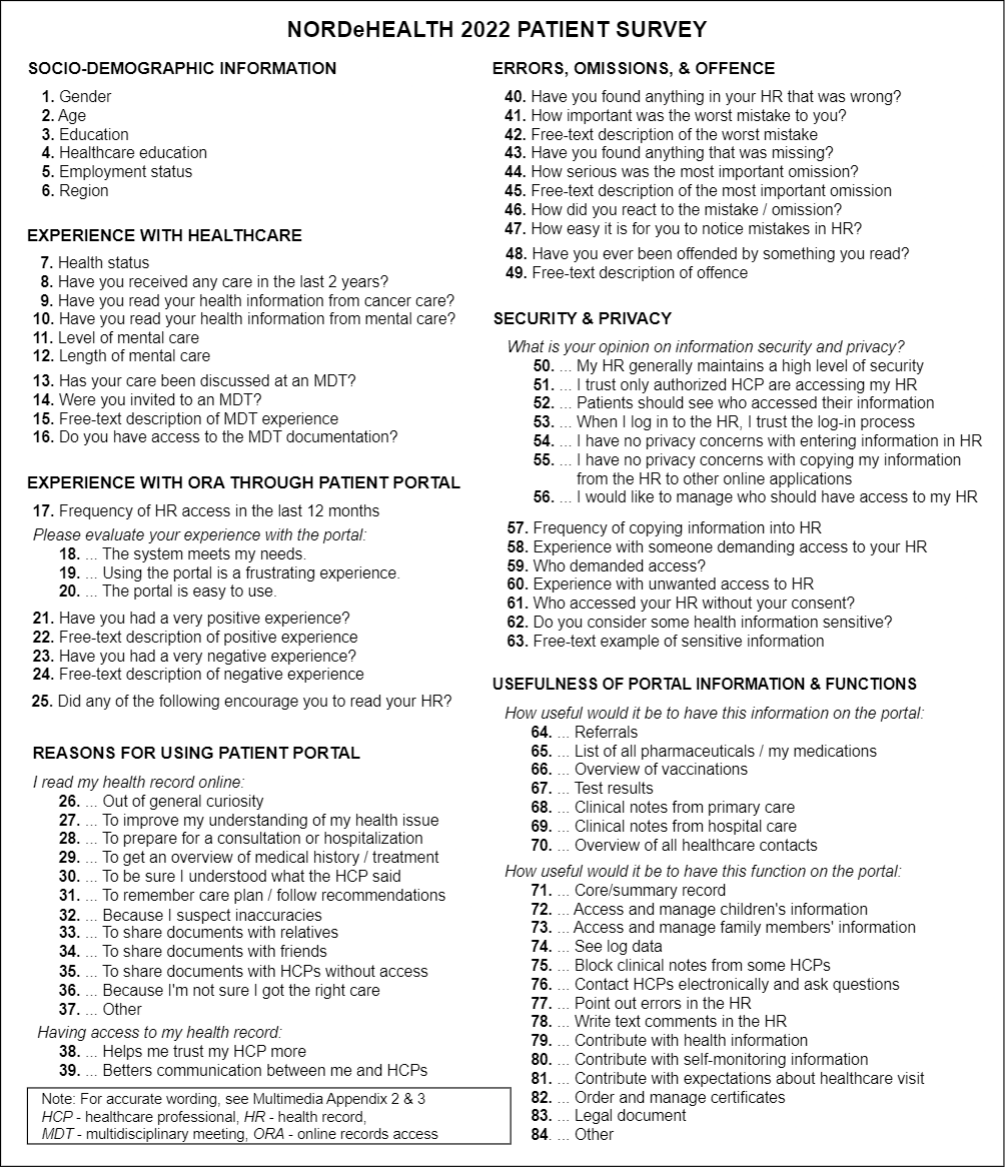
Overview of survey items.

At the beginning of 2022, we conducted a cross-sectional, online anonymous study in Norway, Sweden, Finland, and Estonia, surveying patient users of EHRs available through national patient portals. The survey consisted of a combination of close-ended and free-text questions divided into several thematic sections: (1) sociodemographic information; (2) experience with health care; (3) experience with ORA through the patient portal; (4) reasons for and impact of using the health record; (5) errors, omissions, and offenses; (6) security and privacy; and (7) usefulness of information and functions (Figure 1). The questions were adapted from previous research [10,29,35,36] or newly formulated in line with the current topics of interest. The usability questions (Figure 1: items 19, 20, and 21) have been previously validated and found to be reliable in a health context [40]. The draft survey was created in English and later translated into their respective national languages, resulting in 4 national surveys (Multimedia Appendix 1).

The template survey consisted of 38 closed-ended and 7 free-text questions (Table 1). Of the closed-ended questions, 32 were simple and 6 were compound. Simple questions were self-contained, for example, “Have you ever felt offended by something you read?” while compound questions referred to statements and asked the respondent to evaluate them, for example, “How useful would it be to have access to the following functions in the portal?” referred to statements such as “Ability to access information and manage services for my children.” The exact number of statements differed between countries because of the exclusion of statements that were inapplicable in that country’s context (Multimedia Appendix 1).

**Table 1:** Key similarities and differences between survey structures.

					Norway			Sweden			Finland			Estonia
**Survey structure**														
	Languages			Norwegian			Swedish			Finnish and Swedish^a^			Estonian and Russian^b^	
	Closed-ended questions			Mandatory			Mandatory			Optional			Optional	
	Open-ended items			Optional			Optional			Optional			Optional	
**Number of items^c^**					73 (100)		83 (100)			64 (100)			72 (100)	
	**Closed-ended questions, n (%)**													
		Simple	32 (44)			32 (39)			32 (50)			32 (44)		
		Compound^c^	6			6			5			6		
		Statements			31 (42)			44 (53)			27 (42)			33 (46)
	Free-text questions, n (%)			10 (14)			7 (8)			5 (8)			7 (10)	

^a^98.3% (4639/4719) of the survey was completed in Finnish and 1.7% (80/4719) in Swedish.

^b^81.61% (1717/2104) of the survey was completed in Estonian and 18.4% (387/2104) in Russian.

^c^The total number of survey items does not include closed-ended compound questions in the calculation. No percentage was calculated for compound questions.

Free-text questions also differed in numbers between the national surveys, which was due to how they were presented. In Finland, 2 free-text questions were presented as answer options with free-text entry. In Norway, when an answer option “other” prompted a free-text entry, the text response was input through a separate question that appeared only if the answer option was selected (Multimedia Appendix 1). When counting all survey items that allowed for a participant response (simple closed-ended questions, statements, and free-text questions), the national surveys came to different totals (Table 1).

The answer options for closed-ended questions varied. They included simple “Yes” and “No” answers, ratings on a bipolar anchored Likert scales, for example, a 5-point usefulness scale ranging from 1: “Not useful at all” to 5L “Very useful” and custom categorical answer options, for example, age brackets. Some closed-ended questions allowed only 1 answer, whereas others were multiple-choice questions (Multimedia Appendix 1). Answers to the free-text questions were comments written in the preferred language of the participant. There were no preset character limits restricting the length of the comments.

Some questions were static and presented to all participants, whereas others were dynamic and appeared conditionally based on a previously given answer (Multimedia Appendix 1). This was purposeful to keep the questions relevant to the respondent’s previously given answers. Answering every close-ended question was mandatory for Norway and Sweden but was optional for Finland and Estonia. Free-text questions were optional in all the countries. The draft local versions of the surveys were pretested, and translations were individually adjusted by the national teams where necessary, ahead of the survey launch. The final versions can be viewed in Multimedia Appendix 2.

### Data Collection Strategy

#### Survey Distribution

We aimed to adopt a similar strategy to distribute the survey in each country. For practical reasons, some differences persisted, and these are summarized in Table 2. In all countries, the data collection approach was convenience sampling.

Each national survey was accessible through a link placed on the country’s national patient portal. The Norwegian, Swedish, and Finnish surveys were open for 3 weeks, from January 23, 2022, to February 14, 2022. In Estonia, the survey was open for 9 weeks, from January 23, 2022, to March 28, 2022. This was intentional and motivated by the expected slower data collection rate due to the smaller portal user base as well as survey link placement differences. In Estonia, in the first 2 weeks, the survey link was placed on the page available only after the user had logged out from the portal. Owing to the low response rate, it was moved to the page available directly before logging in. In Finland, the survey link was only presented to the user on the page after logging out. In Norway and Sweden, the survey was accessible only after the patient users authenticated themselves, but the link was placed within the service that provides online access to the EHR, that is, the EHR *Journalen*. In Sweden, the survey link was at the top of the page, while in Norway, the link appeared after the patient user pressed “Done” on reading their record.

**Table 2:** Key similarities and differences between survey distributions.

				Norway		Sweden		Finland		Estonia
**Data collection**										
	Survey platform			Questback		Webropol^a^		Webropol^a^		Decipher
	Length (weeks), n			3		3		3		9
	Advertisement platform			helsenorge.no		1177.se		kanta.fi		digilugu.ee
	Link placement			After log-in		After log-in		After log out		Before log-in and after log out
	Retake prevention			Present		Present		Absent		Present
**Participants**										
	Estimated eligible sample, n			524,209		1,085,092		1,262,708		607,493
	Minimum age requirement (years)			16		15		15		15
	**Clicked on survey link,** **n**			N/A^b^		23,878		5731		11,952
		Dropped out, n (%)	N/A		2859 (11.97)^c^		608 (11.41)		9484 (82.4)	

^a^The surveys in Sweden and Finland were hosted on Webropol but independently of each other.

^b^N/A: not applicable.

^c^The surveys in Norway and Sweden required all close-ended questions to be answered for a participant response to be deemed complete.

Each national team used a separate platform to build and advertise the survey. Inherent differences in platforms led to varying levels of control over who could engage in the survey. In Norway, Sweden, and Estonia, it was not possible to retake the survey as the survey link disappeared once it was clicked while in Finland it was. In Estonia, the survey was carried out by an external company commissioned by the research team (Indico Consulting OÜ) and, in all other countries, by researchers in the NORDeHEALTH project.

To increase the national awareness of the survey, it was advertised in each country across social media and traditional media outlets. Social media platforms included Twitter, Facebook, and LinkedIn. Traditional media included written interviews with the project principal investigator (MH) in national and local newspapers as well as press releases sent by connected universities.

#### Target Population

The target population was patient users from national patient portals who used the service between January and March 2022. Owing to research ethics regulations, the minimum age required to partake was 15 years for all countries except Norway, where participants had to be aged 16 years to use this service. There were 33 participants (Norway, n=2; Sweden, n=2; Finland n=2, Estonia n=27) that reported age below the requirement; thus, they were excluded from the data set and further analysis.

It is not possible to precisely report the number of eligible participants who visited the patient portals while the survey was distributed because of the inability to filter by age. Nonetheless, an estimation was made based on data published by service providers on the total number of individual visits to the national patient portals, or the PAEHR for the duration of the survey advertisement was 524,209 in Norway (visits to the EHR); 1,085,092 in Sweden (visits to the EHR); 1,262,708 in Finland (visits to the national patient portal); and 607,493 in Estonia (visits to the national patient portal).

In Norway and Sweden, it was possible to obtain statistics on the use of the PAEHR service distinctly from the statistics on overall patient portal use. Other functionalities such as appointment bookings and prescription renewals are available on the national patient portals but not within the PAEHR. In contrast, in Estonia and Finland, there is no distinct PAEHR but information from the EHR is available alongside other portal functionality. Hence, use statistics are only available for overall portal use.

### Data Management Strategy

Anonymized data from each country partner were stored securely on a password-protected data platform provided by Uppsala University (Dataportal Allvis), which was approved for storing sensitive research data. Anonymized data sets were shared between countries according to a predetermined data-sharing agreement that lists the data owners for the NORDeHEALTH data set for each country. These are the Norwegian Centre for E-health Research for Norway, Uppsala University for Sweden, Aalto University for Finland, and Tallinn University of Technology for Estonia. Data will be stored at Uppsala University for 10 years in compliance with ethical approvals.

### Ethical Considerations

Before data collection, the survey received ethics approval from the Ethical Review Authority in Sweden (approval #2021-05229), the Aalto University Research Ethics Committee in Finland (approval #D/957/03.04/2020), and the Research Ethics Committee of the National Institute of Health Development in Estonia (protocol #31, approval #977). In Norway, according to the Norwegian Act on Medical and Health Research Section 2 and Section 4, the study did not require approval from the regional ethics committee, but the data-handling procedure was approved by the Data Protection Officer of the University Hospital of North Norway (approval #02799).

### Analysis

To describe the NORDeHEALTH 2022 Patient Survey data set, we analyzed the variables related to participant characteristics (gender, age, education, health care education, and employment) as well as experience with health care and patient portals (health status, care in the last 2 years, and frequency of health record access). Data were summarized using descriptive statistics (count and percentage) per the national sample and for the total data set. Percentages were calculated based on the available data, that is, excluding missing data. The exact amount of missing data for each used variable can be found in Multimedia Appendix 3. Calculations were performed using JASP (version 0.16.4; Amsterdam University). The geographic distribution of the respondents was visualized using a figure built with Datawrapper (Datawrapper GmbH).

## Results

### Participant Characteristics

**Figure 2:**
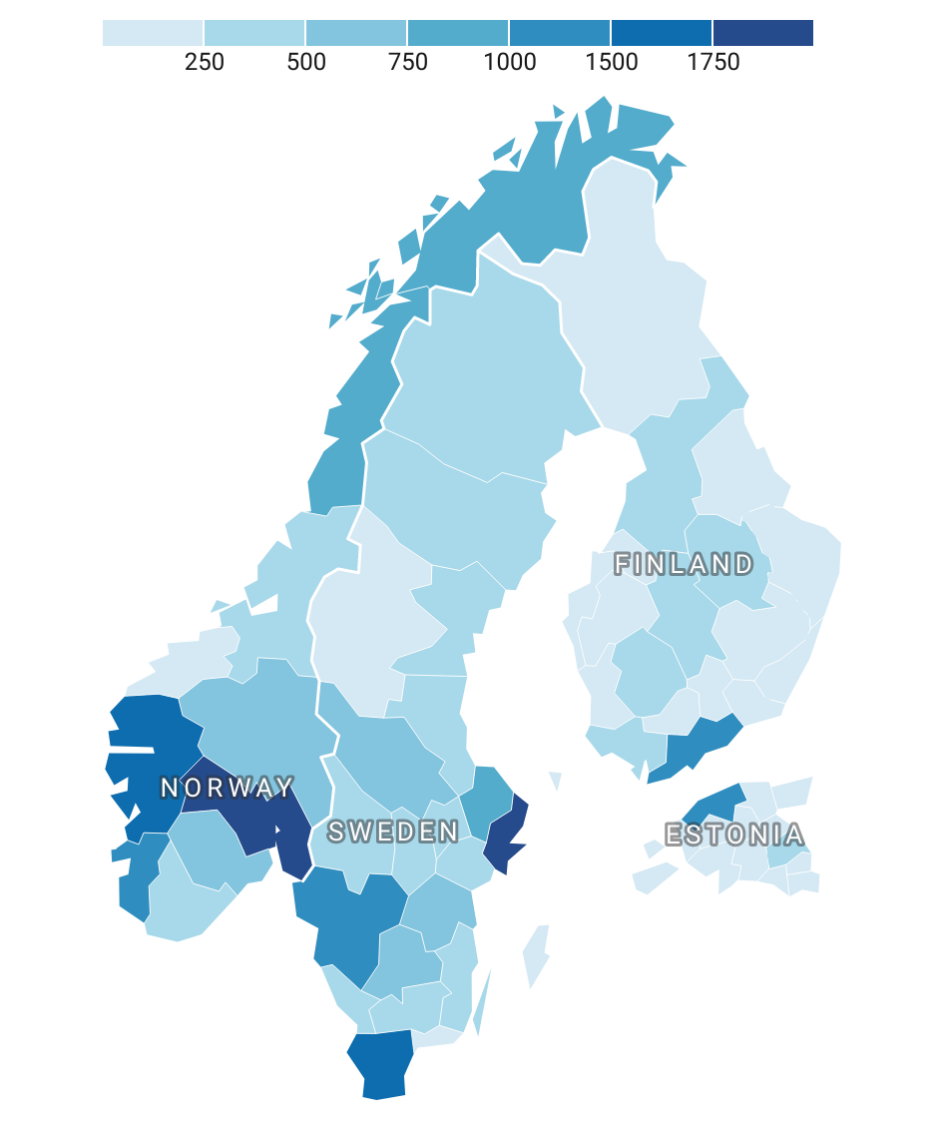
Respondents’ geographic distribution by country and region.

Across the 4 countries, a total of 29,334 respondents completed the survey (Table 3). Almost half of the responses were recorded in Sweden (13,008/29,334, 44.35%), followed by Norway (9503/29,334, 32.4%) and Finland (4719/29,334, 16.07%), with the fewest responses from Estonia (2104/29,334, 7.17%). Estimated from the number of visitors to the patient portal or PAEHR, the response rate in Norway was 1.81% (9503/524,209); in Sweden, it was 1.2% (13,008/1,085,092); in Finland, it was 0.37% (4719/1,262,708); and in Estonia, it was 0.35% (2104/607,493). The geographical distribution of the sample is shown in Figure 2.

Gender proportions were comparable across countries, with two-thirds of the participants identifying as women (19,904/29,302, 67.93%). Those who identified as “Other” were ≤1% for all national samples except Finland, where they constituted 1.32% (62/4708). Almost 60% (17,398/29,305) of the respondents in the whole data set were aged between 45 and 74 years. This was also true for the samples from Norway (5540/9503, 58.3%) and Sweden (7427/13,008, 57.1%). In Estonia, there was a higher proportion of those aged between 35 and 64 years (1298/2100, 61.8%), whereas in Finland, there were more middle-aged and older users aged between 55 and 74 years (2756/4694, 58.71%).

The distribution of education levels differed between countries (Table 3). In the total sample, the largest education group was upper-secondary education (7480/29,251, 25.57%), which was also true for Finland (1217/4645, 26.2%). In Estonia (691/2095, 32.98%) and Sweden (3563/13,008, 27.39%), most had a master’s degree; and in Norway, most had first-cycle higher education (2904/9503, 30.56%). Finland had the smallest proportion of participants with a higher education (1795/4645, 38.64%), which contrasts with Estonia, where 57.85% (1212/2095) of the sample held a higher education degree. The proportion of participants with health care education was comparable between Norway (2533/9503, 26.65%) and Finland (1026/4612, 22.25%), the lowest in Estonia (180/2087, 8.62%), and the highest in Sweden (4299/13,008, 33.05%).

With the exception of Finland, the largest proportion of respondents were engaged in full-time employment in Norway (3860/9503, 40.61%), Sweden (5090/13,008, 39.13%), and Estonia (1215/2104, 57.97%). In Finland, full-time employed participants comprised a quarter of the national sample (1139/4719, 24.26%), whereas the majority were in retirement (2621/4719, 55.83%).

**Table 3:** Participant characteristics^a^.

				Norway (n=9503), n (%)		Sweden (n=13,008), n (%)		Finland (n=4719), n (%)		Estonia (n=2104), n (%)		Total (N=29,334), n (%)
**Gender^b^**												
	Woman			6218 (65.43)		8754 (67.3)		3422 (72.68)		1510 (72.49)		19,904 (67.93)
	Man			3243 (34.13)		4177 (32.11)		1224 (26)		566 (27.17)		9210 (31.43)
	Other			42 (0.44)		77 (0.59)		62 (1.32)		7 (0.34)		188 (0.64)
**Age (years)^c^**												
	15-19			200 (2.1)		218 (1.68)		24 (0.51)		80 (3.81)		522 (1.78)
	20-24			446 (4.69)		409 (3.14)		62 (1.32)		69 (3.29)		986 (3.36)
	25-34			1286 (13.53)		1735 (13.34)		224 (4.77)		335 (15.95)		3580 (12.22)
	35-44			1423 (14.97)		1867 (14.35)		361 (7.69)		457 (21.76)		4108 (14.02)
	45-54			1960 (20.63)		2274 (17.48)		595 (12.68)		443 (21.1)		5272 (17.99)
	55-64			1992 (20.96)		2539 (19.52)		1160 (24.71)		398 (18.95)		6089 (20.78)
	65-74			1588 (16.71)		2614 (20.1)		1596 (34)		239 (11.38)		6037 (20.6)
	75-84			574 (6.04)		1262 (9.7)		620 (13.21)		69 (3.29)		2525 (8.62)
	≥85			34 (0.36)		90 (0.69)		52 (1.11)		10 (0.48)		186 (0.63)
**Education**												
	No formal education			66 (0.69)		76 (0.58)		19 (0.41)		3 (0.14)		164 (0.56)
	Primary education			580 (6.1)		1106 (8.5)		474 (10.2)		112 (5.35)		2272 (7.77)
	Upper-secondary education			2391 (25.16)		3434 (26.4)		1217 (26.2)		438 (20.91)		7480 (25.57)
	Higher vocational education			1192 (12.54)		1978 (15.21)		1033 (22.24)		330 (15.75)		4533 (15.5)
	**Higher education**											
		Bachelor’s	2904 (30.56)		2474 (19.02)		826 (17.78)		475 (22.67)		6679 (22.83)	
		Master’s	2248 (23.66)		3563 (27.39)		886 (19.07)		691 (32.98)		7388 (25.26)	
		Research	122 (1.28)		377 (2.9)		83 (1.79)		46 (2.2)		628 (2.15)	
	Other			—^d^		—		107 (2.30)		—		107 (0.37)
Health care education				2533 (26.65)		4299 (33.05)		1026 (22.25)		180 (8.62)		8038 (27.52)
**Employment**												
	Full-time		3860 (40.62)		5090 (39.13)		1139 (24.26)		1215 (57.97)		11,304 (38.58)	
	Part-time		754 (7.93)		1266 (9.73)		279 (5.94)		217 (10.35)		2516 (8.59)	
	Student		515 (5.42)		708 (5.44)		137 (2.92)		115 (5.49)		1475 (5.03)	
	Retired		1967 (20.7)		4109 (31.59)		2621 (55.83)		278 (13.26)		8975 (30.63)	
	Not able to work		1714 (18.04)		710 (5.46)		160 (3.41)		80 (3.82)		2664 (9.09)	
	Unemployed		161 (1.69)		328 (2.52)		214 (4.56)		104 (4.96)		807 (2.75)	
	Other		532 (5.60)		797 (6.13)		145 (3.09)		87 (4.15)		1561 (5.33)	

^a^Percentages were calculated by excluding missing data.

^b^In Finland, the answer option was “Other/I don’t want to answer.”

^c^In Norway, participants were aged ≥16 years.

^d^In Finland, participants had the additional answer option “Other.”

### Experience With Health Care and Health Records

In total, more than a third of the respondents (11,279/29,302, 38.48%) rated their health status as “fair” (Table 4). This was also the most prevalent rating in the national samples. There was a trend for users to judge their health positively rather than negatively: over a third of the responses in the whole sample were “good” or “very good” (10,498/29,310, 35.82%). Close to half of the Estonian participants judged their health as “good” or “very good” (927/2091, 44.33%), which was the highest of all countries. The Norwegian (2494/9503, 26.24%) and Swedish (3213/13,008, 24.7%) samples had a quarter of users rate their health as “bad” or “very bad.”

Overall, participating patient users sought mental health care more (6214/29,254, 21.24%) than oncological care (3664/29,254, 12.52%). Among the respondents, 86.55% (25,318/29,254) reported receiving treatment for a nonspecified health problem. Norway (2008/9503, 21.13%) and Sweden (3169/13,008, 24.36%) had the highest proportions of participants with mental care experience. The same was true for oncology (Norway: 1433/9503, 15.08%; Sweden: 1719/13,008, 13.22%). Patients who had not received any treatment in the last 2 years were the minority in all countries (1521/293,254, 5.2%), but their proportion in Estonia was the highest (192/2047, 9.38%).

Most participants estimated that they accessed their health record “2 to 9 times” in the last 12 months (11,546/29,306, 39.4%). The most frequent users were from Sweden, where over a third of the sample accessed it “more than 20 times” (4571/13,008, 35.14%). Norway had the highest proportion of first-time users (895/9503, 9.4%).

**Table 4:** Experience with health care and health records^a^.

			Norway (n=9503), n (%)		Sweden (n=13,008), n (%)		Finland (n=4719), n (%)		Estonia (n=2104), n (%)		Total (N=29,334), n (%)
**Health status**											
	Very good	600 (6.31)		1088 (8.36)		187 (3.97)		184 (8.8)		2059 (7.02)	
	Good	2733 (28.75)		3393 (26.08)		1571 (33.37)		743 (35.53)		8440 (28.79)	
	Fair	3204 (33.71)		4992 (38.38)		2233 (47.43)		850 (40.65)		11,279 (38.48)	
	Bad	2126 (22.37)		2625 (20.18)		544 (11.55)		202 (9.66)		5497 (18.75)	
	Very bad	369 (3.88)		588 (4.52)		113 (2.40)		47 (2.25)		1117 (3.81)	
	I do not know/I do not want to answer	473 (4.98)		322 (2.48)		60 (1.27)		65 (3.11)		920 (3.14)	
**Care in the last 2 years^b^**											
	Mental health condition(s)	2008 (21.13)		3169 (24.36)		701 (14.92)		336 (16.41)		6214 (21.24)	
	Oncology	1433 (15.08)		1719 (13.22)		424 (9.03)		88 (4.23)		3664 (12.52)	
	Other health problem(s)	8117 (85.41)		11,142 (86.66)		4289 (91.33)		1770 (86.47)		25,318 (86.55)	
	No treatment	418 (4.4)		674 (5.18)		237 (5.05)		192 (9.38)		1521 (5.2)	
**Frequency of health record access in the last 12 months**											
	Never accessed	—^c^		—		49 (1.04)		23 (1.1)		72 (0.25)	
	First time	895 (9.42)		306 (2.35)		94 (2)		65 (3.1)		1360 (4.64)	
	2-9 times	3562 (37.48)		4694 (36.09)		2354 (50.09)		936 (44.68)		11,546 (39.4)	
	10-20 times	2225 (23.41)		3437 (26.42)		1287 (27.38)		596 (28.45)		7545 (25.75)	
	More than 20 times	2821 (29.69)		4571 (35.14)		916 (19.49)		475 (22.67)		8783 (29.97)	

^a^Percentages were calculated by excluding missing data.

^b^Multiple-choice item: the total will not add up to 100%.

^c^The answer option “Never accessed” was available in Finland and Estonia because it was possible to access the survey through the patient portal without visiting the electronic health record.

## Discussion

### Principal Findings

This paper reports on the first large-scale cross-country survey focusing on patients’ experiences with ORA. The NORDeHEALTH 2022 Patient Survey gathered data from 29,334 patient portal users or PAEHR users from Norway (n=9503, 32.4%), Sweden (n=13,008, 44.35%), Finland (n=4719, 16.07%), and Estonia (n=2104, 7.17%). The national samples were comparable according to reported gender, with two-thirds of the participants identifying as women. Most participants in Norway and Sweden were aged between 45 and 74 years, whereas there were older patient respondents in Finland (aged between 55 and 74 years) and younger respondents in Estonia (aged between 35 and 64 years). The prevalence of younger respondents in Estonia is not surprising, as previous research has found a similar trend in younger patient users [41]. In Finland, the national patient portal is widely adopted by the public, and of the users aged 51 to 65 years, over half have been found to use the portal [42].

Education levels differed among the national patient samples. In the whole data set, a quarter of the respondents had an upper-secondary education. Finland had the smallest proportion of participants with a higher degree (1795/4645, 38.64%) and Estonia had the largest (1212/2095, 57.85%). This is likely related to the respondents’ age. The Finnish patient portal is widely adopted by older patients and is commonly used for prescription renewals, which increase in demand with age. Only a small number of respondents reported having health care education, with the highest proportion being in the Swedish sample (4299/13,008, 33.05%) and the lowest in the Estonian sample (180/2087, 8.62%). The higher proportion in Sweden may be partially explained by the variety of health care education options before university: almost a fifth of the Swedish sample (2406/13,008, 18.45%) had health care training without having a university degree. Employment levels were comparable among Norway, Sweden, and Finland, with most being engaged in full-time employment. In Finland, most participants were in retirement, which is likely related to the higher prevalence of older participants.

When considering patients’ self-rated health status, there was a tendency toward positive ratings, although most rated their health as “fair” in the whole data set (11,279/29,302, 38.48%). Notably, almost half of the Estonian participants (927/2091, 44.33%) rated their health as “good” or “very good.” This trend likely reflects the younger age of the Estonian respondents. Estonia also had the highest number of respondents who had not received any care in the last 2 years (192/2047, 9.38%), with the number being almost double that of the other countries. Respondents from Norway and Sweden reported receiving mental and oncological care the most of all countries and had the most users rate their health as “bad” or “very bad.” Poor self-rated health could partially explain why a third of Sweden’s sample were users who accessed the portal “more than 20 times.” In contrast, Norway had the most first-time users (895/9503, 9.42%), which may be due to the recent implementation of PAEHR in one of the regions. Overall, a few respondents rated their health as “very bad” (1117/29,334, 3.81%). This may be because those with very poor health experience additional barriers related to their health that prevent them from engaging with their EHRs. This would mean that those who may harness the most clinical benefits from ORA may not be active users. However, comparing the Swedish respondents who rated their health as “very bad” or “bad” (3213/13,008, 24.7%) to the corresponding numbers from the Swedish Public Health Survey 2022 where only 6.8% reported their health as being “bad or very bad” [43], the situation is rather the opposite.

### Comparison With Prior Work

Similar surveys have been previously carried out in Norway [35], Sweden [36], Finland [29], and the United States [10], but all of them exclusively collected national data and, apart from the US study, secured smaller sample sizes. The Norwegian survey from 2016 was available for approximately 1 month and gathered 1037 responses [35], which is markedly lower than the present data set from Norway (n=9503). The Swedish survey from 2016 [36] was distributed in the same way as the survey presented in this paper but was open for 5 months and had 2587 respondents compared with the 13,008 responses reported in this study. The increase in the number of users is partially explained by a larger user pool. In 2016, the service was not available in all Swedish regions. Around 0.6 million patients used the Swedish EHR service *Journalen,* compared with 6.8 million today [26]. The Finnish survey from 2021 [29] had 3315 responses compared with the present 4719 but had a higher response rate (0.7% before vs 0.37% in this study), which may be linked to the continued growth in daily patient portal users [28]. Yet, the larger survey samples across countries point to the increased interest in ORA. Because many of the questions in the NORDeHEALTH 2022 Patient Survey were based on Norwegian, Swedish, and Finnish surveys, analyses of change over time will be carried out but are outside the scope of this paper.

The sociodemographic data from the whole sample could be compared with that of the US survey from 2017 [10], but methodological differences should be noted. Unlike this study, the US survey was not distributed nationwide but through 3 health systems that were part of the OpenNotes initiative. Under this initiative, participating institutions provide patients with online access to consultation notes from primary and specialized care [44]. Participants in the US survey were preselected based on whether they had logged into the patient portal at least once in the preceding 12 months and if they had read a consultation note, as indicated by the patient portal data. The inclusion criteria were necessary, as this survey focused on experiences with the consultation notes and not on the overall EHR. This contrasts with the current survey, which used a convenience sampling technique and did not require participants to have previous experiences with EHRs. Furthermore, in the US survey, potential participants were invited through a notification in their portal account or a letter to their personal email. In this study, we placed notifications in the national patient portal, resulting in a larger pool of possible participants and potentially fewer responder biases. Importantly, in the US study, participants were reimbursed for their time while this was not the case in this study. These different strategies may have been responsible for the different response rates: 21.68% (29,656/136,815) responded in the 2017 US Survey. The data gathered in the US study were also linked to the patient portal data, allowing the verification of some of the survey data, such as whether the respondent had previously read any consultation notes. In the present survey, this was not possible because the responses were gathered independently of the patient portal and were anonymous. When comparing the sizes of the data sets, there were 28,782 patient respondents in the US study gathered over 5 months compared with 29,334 gathered over 9 weeks in this study. In both surveys, most respondents were aged ≥45 years, women, and employed. Further focused comparisons with US research would be useful as the digital health care ecosystem and the national portals continue to develop there. The health care systems in the United States and the participating countries in our survey bear considerable differences, which may impact both the design and implementation of patient portals, as well as the patients’ experiences of ORA. By broadening the comparison to the United States and other countries with emerging ORA, we can deepen our understanding of how contextual factors impact the adoption and use of digital patient services.

### Limitations

The cross-national distribution of the survey is one of its leading strengths but some limitations arose with it. The survey was completely anonymous and relied on patients’ self-report instead of gathering some of the information about the user from their profile, similar to that done in other studies [10]. Although the survey template was created in partnership with all the national teams, each national survey was built and distributed independently. This introduced some differences, which should be considered when interpreting the data. In terms of survey structure, it was mandatory to complete all closed-ended questions in Norway and Sweden but not in Finland and Estonia. Free-text questions were optional for all individual surveys. Furthermore, not all questions appeared in all surveys, meaning that the international comparison of some items will not be possible (Multimedia Appendix 1). Regarding the answer options, the Likert scale items had a text label for all answer options in Norway and Finland but not in Sweden and Estonia, where only the anchoring options did. These structural differences arose from a combination of factors owing to the decentralized administration of the survey. Finally, because the template survey was created in English and translated into 5 different languages before distribution, it is likely that there were lingual nuance differences in some of the items. Data collection strategies were largely comparable but had to be adjusted due to advertising on national patient portal platforms. The survey link placement was restricted by the technical implementation of the platform. Most notably, in Estonia, the survey link was moved from the logout page to one after log-in, and the data collection period was considerably extended. Together, these changes boosted the number of responses in Estonia, which had a lower active user base than other countries.

We also note that our data are not representative of the countries’ general populations or of all patient portal users. Previous research shows that women and older users are the most frequent users of digital health services [45], which was also true in our study. Patients who rarely visited the patient portal were likely to miss the narrow period when the survey was available. This should be rectified in future research, where the survey is advertised for longer periods. Patients who did not speak the languages in which the survey was delivered were likely to have been prevented from participating. In the future, such patient surveys should provide more language options or at least an English version. More inclusive answer options for gender as well as questions on race and ethnicity should also be part of the future iterations of the survey. The lack of such data limits the knowledge of minority communities’ experiences with digital health care. In the case of race, omitting the question perpetuates normative whiteness in Nordic research [46].

It should be noted that while these limitations caution the consideration of the survey data in its uniformity, depending on the research question, they do not prevent cross-country comparisons of most of the survey items. They also do not impede analyses within the national samples, many of which are planned.

### Future Directions

Several major analyses of the NORDeHEALTH 2022 Patient Survey data are in progress. They are centered around the project’s work packages [47] as well as topical issues in the respective countries. These include analyses of answers from participants with experience in mental and oncology health care, focused investigations on survey items on security and privacy, multidisciplinary team meetings, and usability of the patient portal [48]. Analyses exploring users of various age groups such as adolescents (aged ≤21 years) and older adults (aged ≥65 years) are also in process. We foresee several reports focused exclusively on national data as well as publications carrying out international comparisons, where applicable. A comparison with previous data was also planned, for example, with the survey carried out in Sweden in 2016 [36].

More broadly, this survey is the first step toward gaining an international perspective on patient portals and patient-accessible EHRs. Future studies should aim to broaden the list of collaborating countries, particularly to countries that are systematically underrepresented in research but that have emerging or established national patient portals and EHRs, such as eHealth (*еЗдраве*) in Bulgaria [49]. Furthermore, a similar international survey aimed at HCPs is needed to gain a comprehensive understanding of all users’ experiences with EHRs. To date, such investigations have only been carried out at the national level [9,50,51]. Finally, while our survey covered a variety of topics related to patient experiences with EHRs, many others were excluded. As indicated by international initiatives, such as the EU Health Data Space [18], there is a growing interest in giving patients the ability to access their health record data abroad as well as contributing to it, for example, through self-monitoring. Research on how users envision the future of patient portals would not only be timely but would also put patients at the core of EHR development.

### Conclusions

The NORDeHEALTH 2022 Patient Survey is the result of the collective effort of experts from Norway, Sweden, Finland, Estonia, and the United States, but it is the first step toward gathering international data on patients’ experiences with EHRs. The planned focused analyses of the data set will provide a unique cross-national perspective on a variety of key aspects that build the patient experience with digital health care.

## Appendix

Multimedia Appendix 1 Template survey and national translations.

Multimedia Appendix 2 Final versions of the national surveys.

Multimedia Appendix 3 The amount of missing data per variable.

## Abbreviations

EHR: electronic health record
EU: European Union
HCP: health care professional
ORA: online record access
PAEHR: patient-accessible electronic health record

## Data Availability

Data are available upon request from MH and following agreement from all data owners.

## References

[1] McCullough JS, Parente ST, Town R (2016). Health information technology and patient outcomes: the role of information and labor coordination. Rand J Econ.

[2] Ossebaard HC, Van Gemert-Pijnen L (2016). eHealth and quality in health care: implementation time. Int J Qual Health Care.

[3] Barello S, Triberti S, Graffigna G, Libreri C, Serino S, Hibbard J, Riva G (2015). eHealth for patient engagement: a systematic review. Front Psychol.

[4] Seddon JJ, Currie WL (2017). Healthcare financialisation and the digital divide in the European Union: narrative and numbers. Inf Manag.

[5] (2020). Digital health platform handbook: building a digital information infrastructure (‎infostructure)‎ for health. World Health Organization.

[6] (2012). Health informatics — personal health records — definition, scope and context: ISO/TR 14292:2012(en). International Organization for Standardization.

[7] Tang PC, Ash JS, Bates DW, Overhage JM, Sands DZ (2006). Personal health records: definitions, benefits, and strategies for overcoming barriers to adoption. J Am Med Inform Assoc.

[8] Wiljer D, Urowitz S, Apatu E, DeLenardo C, Eysenbach G, Harth T, Pai H, Leonard KJ, Canadian Committee for Patient Accessible Health Records (2008). Patient accessible electronic health records: exploring recommendations for successful implementation strategies. J Med Internet Res.

[9] Blease C, Torous J, Dong Z, Davidge G, DesRoches C, Kharko A, Turner A, Jones R, Hägglund M, McMillan B (2023). Patient online record access in English primary care: qualitative survey study of general practitioners' views. J Med Internet Res.

[10] Walker J, Leveille S, Bell S, Chimowitz H, Dong Z, Elmore JG, Fernandez L, Fossa A, Gerard M, Fitzgerald P, Harcourt K, Jackson S, Payne TH, Perez J, Shucard H, Stametz R, DesRoches C, Delbanco T (2019). OpenNotes after 7 years: patient experiences with ongoing access to their clinicians' outpatient visit notes. J Med Internet Res.

[11] Hägglund M, Scandurra I (2017). Patients' online access to electronic health records: current status and experiences from the implementation in Sweden. Stud Health Technol Inform.

[12] Schwarz J, Bärkås A, Blease C, Collins L, Hägglund M, Markham S, Hochwarter S (2021). Sharing clinical notes and electronic health records with people affected by mental health conditions: scoping review. JMIR Ment Health.

[13] Petersson L, Erlingsdóttir G (2018). Open notes in Swedish psychiatric care (Part 1): survey among psychiatric care professionals. JMIR Ment Health.

[14] Kristiansen E, Johansen M, Zanaboni P (2019). Healthcare personnels’ experience with patients’ online access to electronic health records: differences between professions, regions, and somatic and psychiatric healthcare. Proceedings of the 17th Scandinavian Conference on Health Informatics.

[15] Johansen MA, Kummervold PE, Sørensen T, Zanaboni P (2019). Health professionals' experience with patients accessing their electronic health records: results from an online survey. Stud Health Technol Inform.

[16] Hagström J, Scandurra I, Moll J, Blease C, Haage B, Hörhammer I, Hägglund M (2022). Minor and parental access to electronic health records: differences across four countries. Stud Health Technol Inform.

[17] Blease C (2022). Sharing online clinical notes with patients: implications for nocebo effects and health equity. J Med Ethics (Forthcoming).

[18] (2022). A European health data space for people and science. European Commission.

[19] Salmi L, Blease C, Hägglund M, Walker J, DesRoches CM (2021). US policy requires immediate release of records to patients. BMJ.

[20] Turner A, Morris R, McDonagh L, Hamilton F, Blake S, Farr M, Stevenson F, Banks J, Atherton H, Rakhra D, Lasseter G, Feder G, Ziebland S, Hyde E, Powell J, Horwood J (2022). Unintended consequences of patient online access to health records: a qualitative study in UK primary care. Br J Gen Pract.

[21] (2023). The Norwegian Patient Right Act (LOV-1999-07-02-63). Ministry of Health and Welfare, Norway.

[22] (2012). Én innbygger-én journal: Digitale tjenester i helseog omsorgssektoren. Regjeringen.

[23] Direktoratet for e-helse E-helse i tall. Ehelse.no.

[24] Lyttkens CH, Christiansen T, Häkkinen U, Kaarboe O, Sutton M, Welander A (2016). The core of the Nordic health care system is not empty. Nord J Health Econ.

[25] (2022). Befolkningsstatistik. Statistikmyndigheten.

[26] Statistik för 1177 journal. Inera.

[27] Keskimäki L, Tynkkynen LK, Reissell E, Koivusalo M, Syrjä V, Vuorenkoski L, Rechel B, Karanikolos M, World Health Organization, Regional Office for Europe, European Observatory on Health Systems and Policies (2019). Finland: health system review. World Health Organization.

[28] Jormanainen V (2018). Large-scale implementation and adoption of the Finnish national Kanta services in 2010–2017: a prospective, longitudinal, indicator-based study. FinJeHeW.

[29] Kujala S, Hörhammer I, Väyrynen A, Holmroos M, Nättiaho-Rönnholm M, Hägglund M, Johansen MA (2022). Patients' experiences of web-based access to electronic health records in Finland: cross-sectional survey. J Med Internet Res.

[30] Habicht T, Reinap M, Kasekamp K, Sikkut R, Aaben L, Van Ginneken E (2018). Estonia: health system review 2018. World Health Organization (acting as the host organization for, and the Secretariat of, the European Observatory on Health Systems and Policies).

[31] Metsallik J, Ross P, Draheim D, Piho G (2018). Ten years of the e-Health system in Estonia. CEUR Workshop Proceedings.

[32] Tiik M, Ross P (2010). Patient opportunities in the Estonian Electronic Health Record System. Stud Health Technol Inform.

[33] Merimaa K, Vanker E (2020). Terviseportaal eelanalüüs. Ministry of Social Affairs, Estonia.

[34] (2022). Population figure. Statistics Estonia.

[35] Zanaboni P, Kummervold PE, Sørensen T, Johansen MA (2020). Patient use and experience with online access to electronic health records in Norway: results from an online survey. J Med Internet Res.

[36] Moll J, Rexhepi H, Cajander Å, Grünloh C, Huvila I, Hägglund M, Myreteg G, Scandurra I, Åhlfeldt RM (2018). Patients' experiences of accessing their electronic health records: national patient survey in Sweden. J Med Internet Res.

[37] Essén A, Scandurra I, Gerrits R, Humphrey G, Johansen MA, Kierkegaard P, Koskinen J, Liaw ST, Odeh S, Ross P, Ancker JS (2018). Patient access to electronic health records: differences across ten countries. Health Policy Technol.

[38] Home page. NORDeHEALTH.

[39] Hägglund M (2021). Maria Hägglund: Nordic countries lead new initiative on patient access to EHRs. The BMJ.

[40] Borsci S, Buckle P, Walne S (2020). Is the LITE version of the usability metric for user experience (UMUX-LITE) a reliable tool to support rapid assessment of new healthcare technology?. Appl Ergon.

[41] Nøhr C, Parv L, Kink P, Cummings E, Almond H, Nørgaard JR, Turner P (2017). Nationwide citizen access to their health data: analysing and comparing experiences in Denmark, Estonia and Australia. BMC Health Serv Res.

[42] Jormanainen V, Parhiala K, Niemi A, Erhola M, Keskimäki I, Kaila M (2019). Half of the Finnish population accessed their own data: comprehensive access to personal health information online is a corner-stone of digital revolution in Finnish health and social care. FinJeHeW.

[43] Nationella folkhälsoenkäten – Hälsa på lika villkor. Folkhälsomyndigheten.

[44] Bell SK, Delbanco T, Walker J (2017). OpenNotes: how the power of knowing can change health care. NEJM Catal.

[45] Carini E, Villani L, Pezzullo AM, Gentili A, Barbara A, Ricciardi W, Boccia S (2021). The impact of digital patient portals on health outcomes, system efficiency, and patient attitudes: updated systematic literature review. J Med Internet Res.

[46] Rastas A, Essed P, Farquharson K, Pillay K, White EJ (2019). The emergence of race as a social category in Northern Europe. Relating Worlds of Racism : Dehumanisation, Belonging, and the Normativity of European Whiteness.

[47] Project outline. NORDeHEALTH.

[48] Simola S, Hörhammer I, Xu Y, Bärkås A, Fagerlund AJ, Hagström J, Holmroos M, Hägglund M, Johansen MA, Kane B, Kharko A, Scandurra I, Kujala S (2023). Patients' experiences of a national patient portal and its usability: cross-sectional survey study. J Med Internet Res.

[49] (2022). Гражданите вече имат достъп до личното им пациентско досие чрез мобилното приложение „еЗдраве“. Ministry of Health.

[50] Beglaryan M, Petrosyan V, Bunker E (2017). Development of a tripolar model of technology acceptance: hospital-based physicians' perspective on EHR. Int J Med Inform.

[51] Carayon P, Cartmill R, Blosky MA, Brown R, Hackenberg M, Hoonakker P, Hundt AS, Norfolk E, Wetterneck TB, Walker JM (2011). ICU nurses' acceptance of electronic health records. J Am Med Inform Assoc.

