# Supplementary material for: The NORDeHEALTH 2022 Patient Survey: Cross-Sectional Study of National Patient Portal Users in Norway, Sweden, Finland, and Estonia
Source: J Med Internet Res. 2023 Nov 13;25:e47573. doi: 10.2196/47573 (PMC10682922; doi:10.2196/47573)
Supplement: Multimedia Appendix 2 [file jmir_v25i1e47573_app2.pdf]

NORDeHEALTH
2022 Patient Survey

National Surveys

Norway

Pasientjournal på Helsenorge

Målet med denne undersøkelsen er å finne ut hvordan du opplever å ha tilgang til din pasientjournal på internett. De fleste spørsmålene er knyttet til tjenesten Pasientjournal på Helsenorge, hvor du kan lese dokumenter fra behandling eller konsultasjon i spesialisthelsetjenesten. Ved å delta i denne undersøkelsen, bidrar du til at vi bedre forstår når og hvordan pasienter leser journalen sin på nett, og om det er noe som kan gjøres for å bedre opplevelsen av dette.

Det tar 5-10 minutter å svare.

Undersøkelsen er en del av det nordiske forskningsprosjektet NORDeHEALTH, hvor vi vil analysere og sammenligne data fra Norge, Sverige, Finland og Estland. Nasjonalt senter for e-helseforskning er ansvarlig for prosjektet i Norge. Les mer om prosjektet her: https://nordehealth.eu/.

Ingen svar fra denne spørreundersøkelsen kommer til å videreformidles til andre eller til helsepersonell som har skrevet i din pasientjournal. Dersom du er bekymret for mulige feil i din pasientjournal, diskuter det med dem som har skrevet dokumentet.

Undersøkelsen er helt anonym, og det vil ikke være mulig å identifisere deg.

Dine svar er viktige for å kunne videreutvikle tjenesten.

1) * Hva er din alder?

- ○ Under 15 år
- ○ 15-19 år
- ○ 20-24 år
- ○ 25-34 år
- ○ 35-44 år
- ○ 45-54 år
- ○ 55-64 år
- ○ 65-74 år
- ○ 75-84 år
- ○ 85 år eller eldre

2) * Hvor uenig eller enig er du i følgende påstander?

	Helt uenig	Uenig	Litt uenig	Hverken uenig eller enig	Litt enig	Enig	Helt enig	Vet ikke
Pasientjournal på Helsenorge møter mine behov.	○	○	○	○	○	○	○	○
Å bruke Pasientjournal på Helsenorge er en frustrerende opplevelse.	○	○	○	○	○	○	○	○
Pasientjournal på Helsenorge er enkel å bruke.	○	○	○	○	○	○	○	○

3) * Kan du huske å ha hatt en spesielt positiv opplevelse med Pasientjournal på Helsenorge?

○ Ja

○ Nei

Denne informasjonen vises kun i forhåndsvisningen

Følgende betingelser må være oppfylt for at spørsmålet skal vises for respondenten:

Dersom spørsmålet Kan du huske å ha hatt en spesielt positiv opplevelse med Pasientjournal på Helsenorge? inneholder noen av disse alternativene

- Ja

4) Kan du beskrive den positive opplevelsen?

5) * Kan du huske å ha hatt en spesielt negativ opplevelse med Pasientjournal på Helsenorge?

○ Ja

○ Nei

Denne informasjonen vises kun i forhåndsvisningen

Følgende betingelser må være oppfylt for at spørsmålet skal vises for respondenten:

Dersom spørsmålet Kan du huske å ha hatt en spesielt negativ opplevelse med Pasientjournal på Helsenorge? inneholder noen av disse alternativene

- Ja

6) Kan du beskrive den negative opplevelsen?

7) * Hvor mange ganger tror du at du har vært inne og lest pasientjournalen din på Helsenorge de siste 12 månedene?

- Dette er første gangen
- 2-9 ganger
- 10-20 ganger
- Mer enn 20 ganger

8) * Har noen av følgende oppfordret eller minnet deg om å lese pasientjournalen din på Helsenorge? Flere svar er mulig.

- ☐ Helsepersonell
- ☐ Skriftlig informasjon fra sykehus eller andre helseinstitusjoner
- ☐ Familie eller venner
- ☐ Andre pasienter
- ☐ Nettsider, som f.eks. helsenorge.no
- ☐ Aviser, radio, TV, Facebook etc.
- ☐ Ingen har oppfordret meg
- ☐ Annet

Denne informasjonen vises kun i forhåndsvisningen

Følgende betingelser må være oppfylt for at spørsmålet skal vises for respondenten:

Dersom spørsmålet Har noen av følgende oppfordret eller minnet deg om å lese pasientjournalen din på Helsenorge? Flere svar er mulig. inneholder noen av disse alternativene

- Helsepersonell

9) * Hvilke(t) helsepersonell oppfordret eller minnet deg om å gå inn og lese dokumenter i pasientjournalen din? Flere svar er mulig.

☐Lege
☐Sykepleier
☐Psykolog
☐Fysioterapeut
☐Annet helsepersonell (f.eks. helsesekretær)

Denne informasjonen vises kun i forhåndsvisningen

Følgende betingelser må være oppfylt for at spørsmålet skal vises for respondenten:

Dersom spørsmålet Har noen av følgende oppfordret eller minnet deg om å lese pasientjournalen din på Helsenorge? Flere svar er mulig. inneholder noen av disse alternativene

- Annet

Siden du merket av alternativet ANNET for hvordan du har blitt oppfordret til å lese pasientjournalen din på Helsenorge:

10) Kan du beskrive hvem andre eller hvordan du ble oppfordret til å lese pasientjournal (som ikke var listet opp på forrige side)?

11) * Hvor uenig eller enig er du i følgende påstander? Jeg leser pasientjournalen min på Helsenorge ...

	Helt uenig	Uenig	Hverken uenig eller enig	Enig	Helt enig
av nysgjerrighet.	○	○	○	○	○
for å få bedre kunnskap om min egen helse.	○	○	○	○	○
for å forberede meg selv til en konsultasjon eller innleggelse.	○	○	○	○	○

	Helt uenig	Uenig	Hverken uenig eller enig	Enig	Helt enig
for å få oversikt over min sykdomshistorie og/eller behandling.	○	○	○	○	○
for å forsikre meg om at jeg forstod hva legen eller helsepersonellet sa.	○	○	○	○	○
for å huske behandlingsplanen eller følge anbefalingene for behandlingen min.	○	○	○	○	○
fordi jeg mistenker feil, mangler eller unøyaktigheter.	○	○	○	○	○
for å dele dokumenter med familiemedlemmer.	○	○	○	○	○
for å dele dokumenter med venner.	○	○	○	○	○
for å dele dokumenter med helsepersonell som ikke har tilgang til min pasientjournal.	○	○	○	○	○
fordi jeg er usikker på om jeg får riktig behandling.	○	○	○	○	○

12) * Er det andre grunner til at du leser pasientjournalen din på Helsenorge enn de som var listet opp i forrige spørsmål?

○ Nei

○ Ja

Denne informasjonen vises kun i forhåndsvisningen

Følgende betingelser må være oppfylt for at spørsmålet skal vises for respondenten:

Dersom spørsmålet Er det andre grunner til at du leser pasientjournalen din på Helsenorge enn de som var listet opp i forrige spørsmål? inneholder noen av disse alternativene

- Ja

13) Vennligst forklar grunnen(e) til at du leser pasientjournalen din (som ikke var listet opp i forrige punkt).

14) * Hvor uenig eller enig er du i følgende påstander? Å ha tilgang til pasientjournalen min ...

	Helt uenig	Uenig	Hverken uenig eller enig	Enig	Helt enig
bidrar til at jeg stoler mer på min behandler.	○	○	○	○	○
bidrar til bedre kommunikasjon mellom meg og helsepersonellet.	○	○	○	○	○

15) * Har du noen gang lest noe i pasientjournalen din på Helsenorge som du mener ikke var riktig (ikke medregnet feilstavinger og grammatiske feil)?

○ Ja

○ Nei

○ Vet ikke / husker ikke

Denne informasjonen vises kun i forhåndsvisningen

Følgende betingelser må være oppfylt for at spørsmålet skal vises for respondenten:

Dersom spørsmålet Har du noen gang lest noe i pasientjournalen din på Helsenorge som du mener ikke var riktig (ikke medregnet feilstavinger og grammatiske feil)? inneholder noen av disse alternativene

- Ja

16) * Hvor viktig var den alvorligste feilen for deg?

○ Ikke viktig i det hele tatt

○ Litt viktig

○ Svært viktig

○ Usikker

Denne informasjonen vises kun i forhåndsvisningen

Følgende betingelser må være oppfylt for at spørsmålet skal vises for respondenten:

Dersom spørsmålet Har du noen gang lest noe i pasientjournalen din på Helsenorge som du mener ikke var riktig (ikke medregnet feilstavinger og grammatiske feil)? inneholder noen av disse alternativene

- Ja

17) Vennligst beskriv den alvorligste feilen du fant. Ikke inkluder navn eller annen personidentifiserende informasjon.

18) * Har du noen gang oppdaget at noe i pasientjournalen din mangler?

- Ja
- Nei
- Vet ikke / husker ikke

Denne informasjonen vises kun i forhåndsvisningen

Følgende betingelser må være oppfylt for at spørsmålet skal vises for respondenten:

Dersom spørsmålet Har du noen gang oppdaget at noe i pasientjournalen din mangler? inneholder noen av disse alternativene

- Ja

19) * Hvor viktig var den alvorligste mangelen du fant?

- Ikke viktig i det hele tatt
- Litt viktig
- Svært viktig
- Usikker

Denne informasjonen vises kun i forhåndsvisningen

Følgende betingelser må være oppfylt for at spørsmålet skal vises for respondenten:

Dersom spørsmålet Har du noen gang oppdaget at noe i pasientjournalen din mangler? inneholder noen av disse alternativene

- Ja

20) Vennligst beskriv den alvorligste mangelen du fant. Ikke inkluder navn eller annen personidentifiserende informasjon.

Denne informasjonen vises kun i forhåndsvisningen

Følgende betingelser må være oppfylt for at spørsmålet skal vises for respondenten:

Dersom spørsmålet Har du noen gang lest noe i pasientjournalen din på Helsenorge som du mener ikke var riktig (ikke medregnet feilstavinger og grammatiske feil)? inneholder noen av disse alternativene

- Ja

eller

Dersom spørsmålet Har du noen gang oppdaget at noe i pasientjournalen din mangler? inneholder noen av disse alternativene

- Ja

21) * Gjorde du noe av følgende da du oppdaget feilen/mangelen i pasientjournalen din?

- ○ Jeg informerte ansvarlig helsepersonell ved neste besøk.
- ○ Jeg kontaktet det aktuelle helseforetaket/institusjonen via telefon.
- ○ Jeg gjorde ingenting.
- ○ Jeg gjorde noe annet.

Denne informasjonen vises kun i forhåndsvisningen

Følgende betingelser må være oppfylt for at spørsmålet skal vises for respondenten:

Dersom spørsmålet Gjorde du noe av følgende da du oppdaget feilen/mangelen i pasientjournalen din? inneholder noen av disse alternativene

- Jeg gjorde noe annet.

22) Vennligst forklar hva du gjorde da du fant feilen eller mangelen i pasientjournalen din.

23) * Hvor lett (eller vanskelig) er det for deg å oppdage feil /mangler i pasientjournalen din?

- Svært vanskelig
- Vanskelig
- Hverken vanskelig eller lett
- Lett
- Svært lett

24) * Har du noen gang følt deg støtt eller fornærmet på grunn av noe du har lest i pasientjournalen din?

- Ja
- Nei

Denne informasjonen vises kun i forhåndsvisningen

Følgende betingelser må være oppfylt for at spørsmålet skal vises for respondenten:

Dersom spørsmålet Har du noen gang følt deg støtt eller fornærmet på grunn av noe du har lest i pasientjournalen din? inneholder noen av disse alternativene

- Ja

25) Vennligst forklar nærmere hvorfor du har følt deg støtt eller fornærmet. Ikke inkluder navn eller annen personidentifiserende informasjon.

26) * Hvordan vil du beskrive din helsetilstand?

- ◯ Svært god
- ◯ God
- ◯ Nokså god
- ◯ Dårlig
- ◯ Svært dårlig
- ◯ Vet ikke / ønsker ikke å besvare

27) * Har du i løpet av de siste to årene mottatt helsehjelp fra lege (fastlege eller spesialist) eller annet helsepersonell? Flere svar er mulig.

- ☐ Ja, for psykisk helsehjelp
- ☐ Ja, for kreftbehandling
- ☐ Ja, for andre helseproblemer
- ☐ Nei, jeg har ikke mottatt helsehjelp

Denne informasjonen vises kun i forhåndsvisningen

Følgende betingelser må være oppfylt for at spørsmålet skal vises for respondenten:

Dersom spørsmålet Har du i løpet av de siste to årene mottatt helsehjelp fra lege (fastlege eller spesialist) eller annet helsepersonell? Flere svar er mulig. inneholder noen av disse alternativene

- Ja, for psykisk helsehjelp

28) * Har du lest dokumenter i pasientjournalen din på Helsenorge fra psykisk helsehjelp?

- ◯ Jeg har lest alt eller nesten alt.
- ◯ Jeg har lest noe.
- ◯ Jeg har ikke lest noe.

Denne informasjonen vises kun i forhåndsvisningen

Følgende betingelser må være oppfylt for at spørsmålet skal vises for respondenten:

Dersom spørsmålet Har du i løpet av de siste to årene mottatt helsehjelp fra lege (fastlege eller spesialist) eller annet helsepersonell? Flere svar er mulig. inneholder noen av disse alternativene

- Ja, for kreftbehandling

29) * Har du lest dokumenter i pasientjournalen din på Helsenorge fra kreftbehandling?

- Jeg har lest alt eller nesten alt.
- Jeg har lest noe.
- Jeg har ikke lest noe.

Denne informasjonen vises kun i forhåndsvisningen

Følgende betingelser må være oppfylt for at spørsmålet skal vises for respondenten:

Dersom spørsmålet Har du i løpet av de siste to årene mottatt helsehjelp fra lege (fastlege eller spesialist) eller annet helsepersonell? Flere svar er mulig. inneholder noen av disse alternativene

- Ja, for psykisk helsehjelp

30) * Da du mottok psykisk helsehjelp, hvor mottok du den? Flere svar er mulig.

- [ ] Hos fastlegen
- [ ] Dagbehandling/poliklinikk på sykehuset
- [ ] Innleggelse ved sykehuset
- [ ] Gjennom akutt behandling

Denne informasjonen vises kun i forhåndsvisningen

Følgende betingelser må være oppfylt for at spørsmålet skal vises for respondenten:

Dersom spørsmålet Har du i løpet av de siste to årene mottatt helsehjelp fra lege (fastlege eller spesialist) eller annet helsepersonell? Flere svar er mulig. inneholder noen av disse alternativene

- Ja, for psykisk helsehjelp

31) * Hvor lenge har du mottatt/mottok du psykisk helsehjelp?

- Mindre enn 3 måneder
- Mellom 3 måneder og 1 år
- 1-3 år
- Mer enn 3 år

Tverrfaglige team

Tverrfaglige team satt sammen av ulikt helsepersonell brukes i helsetjenesten for å samarbeide rundt behandling av spesifikke sykdommer.

32) * Har noe av din helsebehandling vært diskutert i tverrfaglige team?

- Ja
- Nei
- Vet ikke

Denne informasjonen vises kun i forhåndsvisningen

Følgende betingelser må være oppfylt for at spørsmålet skal vises for respondenten:

Dersom spørsmålet Har noe av din helsebehandling vært diskutert i tverrfaglige team? inneholder noen av disse alternativene

- Ja

33) * Ble du invitert til å delta da det tverrfaglige teamet diskuterte din helsebehandling?

- Ja
- Nei

Denne informasjonen vises kun i forhåndsvisningen

Følgende betingelser må være oppfylt for at spørsmålet skal vises for respondenten:

Dersom spørsmålet Har noe av din helsebehandling vært diskutert i tverrfaglige team? inneholder noen av disse alternativene

- Ja

34) Hva var din opplevelse av det tverrfaglige teamet?

Denne informasjonen vises kun i forhåndsvisningen

Følgende betingelser må være oppfylt for at spørsmålet skal vises for respondenten:

Dersom spørsmålet Har noe av din helsebehandling vært diskutert i tverrfaglige team? inneholder noen av disse alternativene

- Ja

35) * Har du tilgang til dokumentasjon fra møtet i det tverrfaglige teamet?

- Ja
- Nei
- Vet ikke

36) * Hvor uenig eller enig er du i følgende påstander?

	Helt uenig	Uenig	Hverken uenig eller enig	Enig	Helt enig
Jeg synes at min pasientjournal på Helsenorge har et høyt sikkerhetsnivå.					
Jeg stoler på at kun autorisert helsepersonell har tilgang til min pasientjournal.					
Når jeg logger meg på helsenorge.no, stoler jeg på at innloggingsprosessen er sikker.					
Jeg ser ingen sikkerhetsproblemer med å kopiere helseinformasjon fra min pasientjournal til andre internettbaserte programmer eller apper som Google Health, Apple Health, Facebook osv.					

37) * Hvor ofte klipper og limer du inn helseinformasjon fra Pasientjournal på Helsenorge til andre internettbaserte programmer eller apper (som Google Health, Apple Health, Facebook osv.)?

- Jeg har aldri gjort det.
- Jeg har gjort det noen få ganger (1-4 ganger).
- Jeg har gjort det flere ganger (mer enn 5 ganger).

38) * Har du opplevd at familiemedlemmer, venner eller andre har krevd å få tilgang til dokumenter fra Pasientjournal på Helsenorge som du ikke har ønsket å dele?

- Ja
- Nei

○ Vet ikke

Denne informasjonen vises kun i forhåndsvisningen

Følgende betingelser må være oppfylt for at spørsmålet skal vises for respondenten:

Dersom spørsmålet Har du opplevd at familiemedlemmer, venner eller andre har krevd å få tilgang til dokumenter fra Pasientjournal på Helsenorge som du ikke har ønsket å dele? inneholder noen av disse alternativene

- Ja

39) * Hvem krevde å få tilgang til dokumenter som du ikke ønsket å dele?

○ Et familiemedlem

○ En venn

○ Andre

Denne informasjonen vises kun i forhåndsvisningen

Følgende betingelser må være oppfylt for at spørsmålet skal vises for respondenten:

Dersom spørsmålet Hvem krevde å få tilgang til dokumenter som du ikke ønsket å dele? inneholder noen av disse alternativene

- Andre

40) Siden du merket av alternativet ANDRE for hvem som har krevd å få tilgang til dokumenter som du ikke ønsket å dele, ber vi om at du beskriver hvem dette er under. Ikke oppgi navn eller annen personidentifiserbar informasjon.

41) * Har du opplevd at noen har lest dokumenter fra pasientjournalen din på Helsenorge som du ikke ville dele med dem?

○ Ja

○ Nei

○ Vet ikke

Denne informasjonen vises kun i forhåndsvisningen

Følgende betingelser må være oppfylt for at spørsmålet skal vises for respondenten:

Dersom spørsmålet Har du opplevd at noen har lest dokumenter fra pasientjournalen din på Helsenorge som du ikke ville dele med dem? inneholder noen av disse alternativene

- Ja

42) * Hvem leste dokumenter fra pasientjournalen din uten din tillatelse?

- ○ Et helsepersonell
- ○ Et familiemedlem
- ○ En venn
- ○ Andre

Denne informasjonen vises kun i forhåndsvisningen

Følgende betingelser må være oppfylt for at spørsmålet skal vises for respondenten:

Dersom spørsmålet Hvem leste dokumenter fra pasientjournalen din uten din tillatelse? inneholder noen av disse alternativene

- Andre

43) Siden du merket av alternativet ANDRE for hvem som leste dokumenter fra pasientjournalen din uten din tillatelse, ber vi om at du beskriver hvem dette er under. Ikke oppgi navn eller annen personidentifiserbar informasjon.

44) * Anser du enkelte typer helseinformasjon som spesielt sensitiv?

- ○ Ja
- ○ Nei

Denne informasjonen vises kun i forhåndsvisningen

Følgende betingelser må være oppfylt for at spørsmålet skal vises for respondenten:

Dersom spørsmålet Anser du enkelte typer helseinformasjon som spesielt sensitiv? inneholder noen av disse alternativene

- Ja

45) Kan du gi et eksempel på en type helseinformasjon som er spesielt sensitiv for deg?

46) * Hvor nyttig ville det ha vært for deg å ha følgende funksjoner på Helsenorge? Disse funksjonene finnes ikke på Helsenorge i dag.

	Ikke nyttig i det hele tatt	Ikke spesielt nyttig	Hverken ikke nyttig eller nyttig (nøytral)	Nyttig	Svært nyttig
Kontakte helsepersonell elektronisk for å stille spørsmål om innholdet i min pasientjournal	○	○	○	○	○
Påpeke feil i min pasientjournal	○	○	○	○	○
Skrive kommentarer i dokumenter i egen pasientjournal	○	○	○	○	○
Oppdatere egen helsestatus, for eksempel før neste behandling	○	○	○	○	○
Informere om resultater fra selv-tester eller behandling jeg har gjort hjemmefra	○	○	○	○	○
Dele forventninger til neste behandling/kontroll/time	○	○	○	○	○
Bestille eller fornye helseattester eller andre medisinske dokumenter (ikke resepter)	○	○	○	○	○
Få oversikt over alle mine helsekontakter, både i primær- og spesialisthelsetjenesten	○	○	○	○	○
Opprette et juridisk dokument som bekrefter hvilken	○	○	○	○	○

	Ikke nyttig i det hele tatt	Ikke spesielt nyttig	Hverken ikke nyttig eller nyttig (nøytral)	Nyttig	Svært nyttig
medisinsk behandling jeg ønsker dersom jeg havner i en situasjon der jeg ikke kan kommunisere					
Lese min pasientjournal fra fastlegen på nett	◌	◌	◌	◌	◌

Bakgrunnsinformasjon

47) * Hvilket fylke bor du i?

- ◌ Agder
- ◌ Innlandet
- ◌ Møre og Romsdal
- ◌ Nordland
- ◌ Oslo
- ◌ Rogaland
- ◌ Vestfold og Telemark
- ◌ Troms og Finnmark
- ◌ Trøndelag
- ◌ Vestland
- ◌ Viken
- ◌ Annet. Vennligst forklar.

48) * Hvilket kjønn er du?

- ◌ Kvinne
- ◌ Mann
- ◌ Annet

49) * Hva er din høyeste fullførte utdanning?

- ◌ Ingen formell utdanning
- ◌ Grunnskole
- ◌ Videregående skole

- Fagskole
- Høyere utdanning, 2-4 års varighet
- Høyere utdanning, mer enn 4 års varighet
- Doktorgrad

50) * Har du helsefaglig utdanning?

- Ja
- Nei

51) * Hva beskriver ditt arbeidsforhold best? Velg den mest relevante.

- Fulltid
- Deltid
- Student
- Pensjonist
- Arbeidsledig
- Ufør
- Annet

Skapt med Questback Experience Management
Gratis prøveabonnement – lag en spørreundersøkelse med Questback

Sweden

Enkät om din användning och upplevelser av att läsa din journal via nätet

Det nordiska forskningsprojektet NORDeHEALTH syftar till att utforska hur patienter i allmänhet, och inom psykiatri och onkologi i synnerhet, upplever tillgången till sin journal via nätet. Vi vill ta reda på både vilken nytta det ger patienter att ha tillgång till sin information, och vilka utmaningar eller brister det finns. Genom att delta i studien kan du hjälpa oss att förstå vad som behöver förbättras med dagens system. I projektet kommer vi dels analysera resultaten från Sverige, och dels jämföra dessa med resultat från Norge, Finland och Estland.

Enkäten besvaras anonymt, men vi kommer att ställa bakgrundsfrågor om dig (t ex kön, ålder, utbildningsnivå, om du har fått vård för psykiatriska besvär eller cancer) för att kunna jämföra hur människor med olika bakgrund och erfarenhet upplever ”Journalen”. Dina svar kommer att behandlas så att inte obehöriga kan ta del av dem. Studien är helt frivillig att delta i och du kan när som helst avbryta studien utan att ange en orsak.
Du kan läsa mer om hur dina enkätsvar hanteras här (ladda gärna ner och spara informationen).

Om du har några frågor om studien kan du kontakta ansvarig forskare, Maria Hägglund, Uppsala universitet (maria.hagglund@kbh.uu.se alt 0729 999 381).
Om enkäten väcker frågor eller tankar om din vård och hälsa, eller innehållet i din journal, ber vi dig kontakta din vårdgivare.

1. Jag har fått skriftlig information om studien och har haft möjlighet att ställa frågor. Jag får behålla den skriftliga informationen. *

- ○ Jag samtycker till att delta i studien och till att uppgifter om mig behandlas på det sätt som beskrivs i informationen.

2. Ålder: *

- ○ 14 eller yngre
- ○ 15 - 19 år
- ○ 20 - 24 år
- ○ 25 - 34 år
- ○ 35 - 44 år
- ○ 45 - 54 år
- ○ 55 - 64 år
- ○ 65 - 74 år
- ○ 75 - 84 år
- ○ 85 år eller äldre

Du har angett att du är 14 år eller yngre. För att svara på den här enkäten behöver du vara minst 15 år. Enkäten kommer därför att avslutas när du klickar på "Nästa".
Tack för visat intresse!

Användbarhet

3. Utvärdera din upplevelse av att använda Journalen. *

	1. Instämmer inte alls	2	3	4	5	6	7. Instämmer helt	8. Jag vet inte
Journalen tillgodoser mina behov	◯	◯	◯	◯	◯	◯	◯	◯
Att använda Journalen är en frustrerande upplevelse.	◯	◯	◯	◯	◯	◯	◯	◯
Journalen är lätt att använda.	◯	◯	◯	◯	◯	◯	◯	◯

4. Har du haft någon särskilt positiv upplevelse av Journalen? *

◯ Ja
◯ Nej

5. Beskriv den positiva erfarenheten så tydligt som möjligt.

6. Har du haft någon särskilt negativ erfarenhet av Journalen? *

◯ Ja
◯ Nej

7. Beskriv den negativa erfarenheten så tydligt som möjligt.

Följande frågor rör dina erfarenheter av att logga in och läsa din journal.

8. Hur ofta har du läst i din journal under de senaste 12 månaderna? *

- ◯ Det här är första gången
- ◯ 2-9 gånger
- ◯ 10-20 gånger
- ◯ Mer än 20 gånger

9. Har du blivit uppmuntrad eller påmind att läsa din journal av någon/något av följande: *

Markera alla svar som stämmer för dig.

- ☐ Vårdpersonal
- ☐ Läkare
- ☐ Sjuksköterska
- ☐ Psykolog
- ☐ Fysioterapeut/sjukgymnast
- ☐ Annan vårdpersonal
- ☐ Skriftlig information från sjukhuset/vårdinrättning/klinik
- ☐ Familj eller vänner
- ☐ Webbsidor, såsom 1177.se Vårdguiden, etc.
- ☐ Tidningar, radio, TV, Facebook, etc.
- ☐ Andra patienter
- ☐ Ingen har uppmuntrat eller påmint mig
- ☐ Annat:

10. Markera i vilken utsträckning du instämmer med följande påståenden:

Jag läser min journal på internet...

	1. Instämmer inte	2	3	4	5. Instämmer
Av nyfikenhet *	○	○	○	○	○
För att förbättra min förståelse för mitt hälsotillstånd *	○	○	○	○	○
För att förbereda mig själv för ett vårdbesök eller sjukhusvistelse *	○	○	○	○	○
För att få en överblick av min hälsohistoria och/eller bakgrund *	○	○	○	○	○
För att försäkra mig om att jag förstått vad läkaren/vårdpersonalen sagt *	○	○	○	○	○
För att komma ihåg vårdplanen/följa mina behandlingsrekommendationer *	○	○	○	○	○
För att jag misstänker felaktigheter *	○	○	○	○	○
För att dela med mig av information till släkt *	○	○	○	○	○
För att dela med mig av information till vänner *	○	○	○	○	○
För att dela med mig av information till vårdpersonal som inte har tillgång till min journal *	○	○	○	○	○
För att jag är osäker på om jag fått rätt vård *	○	○	○	○	○
Annat, specificera gärna: ____________	○	○	○	○	○

11. Markera i vilken utsträckning du instämmer med följande påståenden: *

Att ha tillgång till min journal...

	1. Instämmer inte	2	3	4	5. Instämmer
Ökar min tillit till mina vårdgivare	○	○	○	○	○
Stödjer bättre kommunikationen mellan mig och vårdpersonal	○	○	○	○	○

12. Har du någonsin stött på felaktigheter i din journal (inte inräknat stavfel eller typografiska fel)? *

○ Ja

○ Nej

○ Vet inte/kommer inte ihåg

13. Hur viktig var den mest allvarliga felaktigheten för dig? *

○ Inte alls viktig

○ Något viktig

○ Väldigt viktig

○ Jag är osäker

Beskriv den mest allvarliga felaktighet du hittat. (inkludera inte namn, information som kan identifiera personer eller känslig information)
Vi kommer inte rapportera detta (eller någon annan information i den här enkäten) till dina vårdgivare. Om du är orolig över en möjlig felaktighet, kontakta din vårdgivare.

14. Har du någonsin stött på att viktig information saknas i din journal? *

○ Ja

○ Nej

○ Vet inte/kommer inte ihåg

15. Hur viktig var den mest allvarliga saknade informationen för dig? *

○ Inte alls viktig

○ Något viktig

○ Väldigt viktig

○ Jag är osäker

Beskriv den mest allvarliga saknade informationen. (inkludera inte namn, information som kan identifiera personer eller känslig information)
Vi kommer inte rapportera detta (eller någon annan information i den här enkäten) till dina vårdgivare. Om du är orolig över en möjlig felaktighet, kontakta din vårdgivare.

16. Gjorde du något av följande när du stötte på felaktigheter eller saknad information i din journal? *

Välj det viktigaste alternativet.

- ◯ Informerade vårdpersonalen vid nästa besök
- ◯ Kontaktade vårdinrättningen över telefon
- ◯ Gjorde inte något
- ◯ Gjorde något annat:

17. Hur lätt (eller svårt) är det för dig att hitta felaktigheter i din journal? *

◯ 1. Väldigt svårt ◯ 2 ◯ 3 ◯ 4 ◯ 5. Väldigt lätt

18. Har du någonsin känt dig förolämpad/sårad/illa berörd av något du läst i din journal? *

- ◯ Ja
- ◯ Nej

19. Förklara gärna.

Följande frågor handlar om din hälsa, vården som du har fått och information som du fått i Journalen om vården.

20. Hur är din allmänna hälsa? *

- ◯ Väldigt bra
- ◯ Bra
- ◯ Ganska bra
- ◯ Dålig
- ◯ Väldigt dålig
- ◯ Jag vet inte/jag vill inte svara

21. Under de senaste 2 åren, har du varit i kontakt med läkare (primärvårdsläkare eller specialist) eller annan vårdpersonal för: *

Markera alla svar som stämmer för dig, flera val är möjligt.

- ☐ Psykisk ohälsa
- ☐ Cancer
- ☐ Andra hälsoproblem
- ☐ Jag har inte fått någon vård/behandling

22. Har du läst information om din vård för psykisk ohälsa online i tjänsten Journalen? *

- ◯ Jag har läst allt/nästan allt i Journalen
- ◯ Jag har läst delar av Journalen
- ◯ Jag har inte läst i Journalen

23. När du fick vård för psykisk ohälsa, på vilken vårdnivå ägde detta rum? *

Markera alla svar som stämmer för dig, flera val är möjligt.

- ☐ Primärvård (vårdcentral)
- ☐ Psykiatri öppenvård (specialistkonsultation, ej inlagd)
- ☐ Psykiatri slutenvård (inlagd)
- ☐ Akutsjukvård

24. Hur länge har du fått vård för psykisk ohälsa? *

- Mindre än 3 månader
- 3 månader - 1 år
- 1-3 år
- Längre än 3 år

25. Har du läst information om din vård för cancer online i tjänsten Journalen? *

- Jag har läst allt/nästan allt i Journalen
- Jag har läst delar av Journalen
- Jag har inte läst i Journalen

Multidisciplinära konferenser används ibland i sjukvården, i situationer för samverkan kring specifika sjukdomar. I konferenserna medverkar olika specialister, beroende på den diagnos som ska diskuteras.

26. Har din vård diskuterats vid en multidisciplinär konferens (MDK)? *

- Ja
- Nej
- Jag vet inte

27. Var du inbjuden på MDK-mötet när din vård diskuterades? *

- Ja
- Nej

28. Vad var din upplevelse av MDK-mötet?

____

____

____

____

____

29. Har du tillgång till dokumentationen från MDK-mötet? *

- Ja

Nej

Vet inte

30. Vad tycker du om sekretess och informationssäkerhet? *

Markera i vilken utsträckning du instämmer med följande påståenden.

	1. Instämmer inte	2	3	4	5. Instämmer
Jag upplever att Journalen generellt håller en hög säkerhetsnivå.					
Jag litar på att endast behörig vårdpersonal har tillgång till min journal.					
Patienter borde kunna se vem som har läst deras patientinformation.					
När jag loggar in i Journalen litar jag på att inloggningen sker på ett säkert sätt.					
Utifrån min personliga integritet har jag inga problem med att kunna lägga till ytterligare hälsoinformation från mina egna källor i Journalen, till exempel träningsdata, mätvärden (som t.ex. vikt, blodtryck) och andra hälsodata från privata appar.					
Utifrån min personliga integritet har jag inga problem med att kopiera min hälsoinformation från Journalen till andra appar online (t.ex. Google Health, Apple Health, Facebook etc.)					
Jag skulle själv vilja ha möjlighet att hantera vem som har tillgång till min journal (till exempel att en särskild diagnos inte kan ses av viss vårdpersonal eller närstående).					

31. Hur ofta kopierar och klistrar du in information från din journal i andra online-appar (Google Health, Apple Health, Facebook etc)? *

Jag har aldrig delat med mig av information

Jag har delat med mig av information några gånger (1-4 gånger)

Jag har delat med mig av information många gånger (mer än 5 gånger)

32. Har du varit med om att din familj, vänner eller andra har begärt tillgång till delar av din journal som du inte velat dela med dig av? *

Ja

- Nej
- Jag vet inte

33. Vem begärde tillgång till din journal som du inte ville dela med dig av? *

- En familjemedlem
- En vän
- Annan: ______________________

34. Har du varit med om att någon har läst delar av din journal som du inte velat dela med dig av? *

- Ja
- Nej
- Jag vet inte

35. Vem läste i din journal utan ditt samtycke? *

- Vårdpersonal
- En familjemedlem
- En vän
- Annan: ______________________

36. Anser du att vissa typer av hälsoinformation är särskilt känsliga? *

- Ja
- Nej

37. Kan du ge ett exempel på vilken typ av hälsoinformation som är mest känslig för dig?

____

____

____

38. Hur användbart skulle det vara för dig att ha tillgång till följande information i Journalen? *

Denna information finns inte i alla regioner idag.

	Inte alls användbart	Inte särskilt användbart	Varken eller	Användbart	Väldigt användbart
En sammanfattning av journalen med viktig information	○	○	○	○	○
Remisser (innehåll och status)	○	○	○	○	○
Lista över mina läkemedel/mediciner	○	○	○	○	○
Vaccinationer	○	○	○	○	○
Provsvar	○	○	○	○	○
Journalanteckningar från primärvården	○	○	○	○	○
Journalanteckningar från specialistvården	○	○	○	○	○
Överblick av samtliga kontakter med vården	○	○	○	○	○

39. Hur användbart skulle det vara för dig att ha tillgång till följande funktioner i Journalen?

Funktionerna finns inte i alla regioner idag. Om du har tillgång till funktionen idag - skatta hur användbar den är för dig.

	Inte alls användbart	Inte särskilt användbart	Varken eller	Användbart	Väldigt användbart
Möjlighet att ta del av information och hantera tjänster för mina barn *	○	○	○	○	○
Möjlighet att ta del av information och hantera tjänster för andra familjemedlemmar *	○	○	○	○	○
Möjlighet att kunna blockera specifika journalanteckningar för viss vårdpersonal *	○	○	○	○	○
Se vilka vårdinrättningar och personal som har läst i min journal (loggar) *	○	○	○	○	○
Möjlighet att kunna kontakta vårdpersonal online för att ställa frågor om min journal *	○	○	○	○	○
Möjlighet att påpeka felaktigheter i journalen *	○	○	○	○	○

	Inte alls användbart	Inte särskilt användbart	Varken eller	Användbart	Väldigt användbart
Möjlighet att skriva egna kommentarer i journalen *	◯	◯	◯	◯	◯
Möjlighet att bidra med information om min hälsa, t ex genom att fylla i en hälsodeklaration, inför ett besök *	◯	◯	◯	◯	◯
Möjlighet att bidra med information från självtest/monitorering hemma *	◯	◯	◯	◯	◯
Möjlighet att bidra med information om mina förväntningar på vårdbesöket *	◯	◯	◯	◯	◯
Möjlighet att beställa och hantera sjukskrivning, medicinsk intyg och andra dokument (vaccinationsintyg t ex) *	◯	◯	◯	◯	◯
Annat ____________	◯	◯	◯	◯	◯

Bakgrundsinformation

40. Region där din vård huvudsakligen sker: *

- ◯ Blekinge
- ◯ Dalarna
- ◯ Gotland
- ◯ Gävleborg
- ◯ Halland
- ◯ Jämtland Härjedalen
- ◯ Jönköpings län
- ◯ Kalmar län
- ◯ Kronoberg
- ◯ Norrbotten
- ◯ Skåne
- ◯ Stockholms län
- ◯ Sörmland
- ◯ Uppsala län
- ◯ Värmland
- ◯ Västerbotten
- ◯ Västernorrland
- ◯ Västmanland
- ◯ Västra Götaland

- Örebro län
- Östergötland
- Vill inte uppge

41. Kön: *

- Kvinna
- Man
- Annat

42. Vilken är din högsta avslutade utbildning? *

- Ingen formell utbildning
- Grundskola eller motsvarande
- Gymnasieexamen eller motsvarande
- Eftergymnasial utbildning, ej högskola/universitet
- Högskole-/universitetsutbildning, 3 år eller mindre
- Högskole-/universitetsutbildning, mer än 3 år
- Utbildning på forskarnivå (doktor, licentiat)

43. Har du utbildning inom vården? *

- Ja
- Nej

44. Vilket av följande beskriver bäst din sysselsättning för närvarande? Välj det svar som passar dig bäst. *

- Heltidsarbete
- Deltidsarbete
- Student
- Pensionär
- Arbetslös
- Kan inte arbeta
- Inget av ovan

Finland

Finnish

Finnish

Ikäsi

1 = 14 vuotta tai alle

2 = 15 – 17 vuotta

3 = 18 – 19 vuotta

4 = 20 – 24 vuotta

5 = 25 – 34 vuotta

6 = 35 – 44 vuotta

7 = 45 – 54 vuotta

8 = 55 – 64 vuotta

9 = 65 – 74 vuotta

10 = 75 – 84 vuotta

11 = 85 vuotta tai enemmän

Arvioi kokemuksesi perusteella Omakantaa (1 = Täysin eri mieltä, 7 = Täysin samaa mieltä)

Omakannan ominaisuudet vastaavat tarpeitani

Omakannan käyttäminen on turhauttava kokemus

Omakantaa on helppo käyttää

Onko sinulla jokin erityisen positiivinen kokemus Omakannan käytöstä?

1 = Kyllä

2 = Ei

Onko sinulla jokin erityisen negatiivinen kokemus Omakannan käytöstä?

1 = Kyllä

2 = Ei

Kuinka usein viimeisen 12 kuukauden aikana olet käynyt katsomassa hoidostasi tehtyjä merkintöjä Omakannasta? (Valitse sopivin vaihtoehto)

1 = En yhtään kertaa

2 = Tämä on ensimmäinen kertani

3 = 2-9 kertaa

4 = 10-20 kertaa

5 = Yli 20 kertaa

Onko jokin seuraavista ohjannut tai muistuttanut sinua katsomaan hoidostasi tehtyjä merkintöjä Omakannasta? (Valitse kaikki sopivat vaihtoehdot)

1 = Lääkäri

2 = Hoitaja

3 = Psykologi

4 = Fysioterapeutti

5 = Muu terveydenhuollon ammattilainen

6 = Kirjallinen ohje terveydenhuollosta

7 = Perhe tai ystävät

8 = Muut potilaat

9 = Kanta.fi tai muu virallinen terveyssivusto

10 = Sanomalehdet, radio, TV tai sosiaalinen media

11 = Muu, mikä?

12 = Ei mikään ylläolevista

Mistä syystä katsot hoidostasi tehtyjä merkintöjä Omakannasta? Ota kantaa seuraaviin väittämiin.

1 = Täysin eri mieltä

2 = Melko eri mieltä

3 = Ei samaa eikä eri mieltä

4 = Melko samaa mieltä

5 = Täysin samaa mieltä

Yleisestä mielenkiinnosta

Parantaakseni ymmärrystäni omasta terveydentilastani

Valmistautuakseni lääkärikäyntiä tai sairaalahoitoa varten

Tarkastellakseni hoitohistoriaani

Varmistaakseni, että ymmärsin, mitä lääkäri sanoi

Seuratakseni saamiani hoito-ohjeita

Koska epäilen, että tiedoissa on virheitä tai puutteita

Jakaakseni tietojani sukulaisille

Jakaakseni tietojani ystäville

Jakaakseni tietojani muulle ammattilaiselle tai viranomaiselle, joilla ei ole pääsyä tietoihin

Koska en ole varma, sainko oikeaa hoitoa

Mitä mieltä olet siitä, että pääset halutessasi näkemään hoidostasi tehdyt merkinnät? Ota kantaa seuraaviin väittämiin.

1 = Täysin eri mieltä

2 = Melko eri mieltä

3 = Ei samaa eikä eri mieltä

4 = Melko samaa mieltä

5 = Täysin samaa mieltä

Pääsy lisää luottamustani terveydenhuoltoon

Pääsy tukee minun ja terveydenhuollon ammattilaisten välistä vuorovaikutusta

Oletko joskus havainnut virheen hoidostasi tehdyissä merkinnöissä (jos kirjoitus- ja näppäilyvirheitä ei huomioida)?

1 = Kyllä

2 = Ei

3 = En tiedä / en muista

Kuinka vakava oli pahin havaitsemasi virhe?

1 = Ei lainkaan vakava

2 = Jokseenkin vakava

3 = Erittäin vakava

4 = En ole varma

Oletko joskus havainnut puuttuvia tietoja hoidostasi tehdyissä merkinnöissä?

1 = Kyllä

2 = Ei

3 = En tiedä / en muista

Kuinka vakava oli pahin tiedon puuttuminen?

1 = Ei lainkaan vakava

2 = Jokseenkin vakava

3 = Erittäin vakava

4 = En ole varma

Teitkö jotain seuraavista, kun havaitsit virheen tai puuttuvan tiedon?

1 = Kerroin tästä terveydenhuollon ammattilaiselle seuraavalla käynnillä

2 = Otin yhteyttä terveydenhuoltoon puhelimitse

3 = En tehnyt mitään

4 = Jotain muuta, mitä?

Kuinka helppoa sinun on havaita virheitä tai puuttuvia tietoja hoidostasi tehdyissä merkinnöissä?

1 = Erittäin vaikeaa

2 = Vaikeaa

3 = Ei vaikeaa eikä helppoa

4 = Helppoa

5 = Erittäin helppoa

Oletko kokenut jonkin Omakannasta lukemasi asian loukkaavana?

1 = En

2 = Kyllä, miten?

Onko terveydentilasi mielestäsi nykyisin:

1 = Erittäin hyvä

2 = Hyvä

3 = Kohtalainen

4 = Huono

5 = Erittäin huono

6 = En tiedä tai en halua kertoa

Oletko saanut lääkärin tai muun terveydenhuollon ammattilaisen hoitoa viimeisen kahden (2) vuoden aikana? (Valitse kaikki sopivat vaihtoehdot.)

1 = Kyllä, mielenterveyteen

2 = Kyllä, syöpäsairauksiin

3 = Kyllä, muihin terveysongelmiin

4 = En ole saanut hoitoa

Oletko lukenut mielenterveyden hoitoasi koskevia tietoja Omakannasta?

1 = Kyllä, olen lukenut kaikki tai lähes kaikki tiedot

2 = Kyllä, olen lukenut tiedot osittain

3 = En ole lukenut tietoja

Oletko lukenut syöpähoitoasi koskevia tietoja Omakannasta?

1 = Kyllä, olen lukenut kaikki tai lähes kaikki tiedot

2 = Kyllä, olen lukenut tiedot osittain

3 = En ole lukenut tietoja

Missä olet saanut hoitoa mielenterveyteen? (Valitse kaikki sopivat vaihtoehdot)

1 = Terveyskeskus, muu kunnallinen tai yksityinen hoitopiste (esimerkiksi psykoterapia)

2 = Sairaalan avohoito (erikoislääkärin konsultaatio)

3 = Sairaalan osastohoito

4 = Sairaalan päivystys

Kuinka pitkään olet saanut hoitoa mielenterveyteen?

1 = Alle 3 kuukautta

2 = 3-12 kuukautta

3 = 1-3 vuotta

4 = Yli 3 vuotta

Onko hoidostasi keskusteltu moniammatillisen tiimin kokouksessa?

1 = Kyllä

2 = Ei

3 = En tiedä

Oliko sinut kutsuttu mukaan moniammatillisen tiimin kokoukseen, kun hoidostasi keskusteltiin?

1 = Kyllä

2 = Ei

Onko sinulla pääsy moniammatillisen tiimin kokouksen muistioihin?

1 = Kyllä

2 = Ei

3 = En tiedä

Kuinka samaa mieltä olet seuraavista väitteistä?

1 = Täysin eri mieltä

2 = Melko eri mieltä

3 = Ei samaa eikä eri mieltä

4 = Melko samaa mieltä

5 = Täysin samaa mieltä

Luotan siihen, että vain valtuutetut terveydenhuollon työntekijät katsovat terveystietojani

Haluan pystyä hallinnoimaan, kuka pääsee katsomaan terveystietojani (esim. jokin käyntitieto ei ole näkyvissä)

Mielestäni Omakanta on tietoturvallinen Luotan, että kirjautuminen Omakantaan on turvallista

Oletko kopioinut tietojasi Omakannasta verkkosovelluksiin (esim. Google Health tai Facebook)?

1 = En ole koskaan kopioinut tietoa

2 = Olen kopioinut tietoa muutaman kerran (1–4 kertaa)

3 = Olen kopioinut tietoa usein (yli 5 kertaa)

Oletko kokenut, että joku on vaatinut päästä katsomaan hoidostasi tehtyjä merkintöjä, joita et olisi halunnut jakaa?

1 = Kyllä

2 = En

3 = En tiedä

Kuka on vaatinut päästä katsomaan hoidostasi tehtyjä merkintöjä, joita et olisi halunnut jakaa?

1 = Perheenjäsen

2 = Ystävä

3 = Muu, kuka?

Oletko kokenut, että joku on nähnyt hoidostasi tehtyjä merkintöjä, joita et olisi halunnut jakaa?

1 = Kyllä

2 = En

3 = En tiedä

Kuka on nähnyt hoidostasi tehtyjä merkintöjä, joita et olisi halunnut jakaa?

1 = Terveydenhuollon ammattilainen

2 = Perheenjäsen

3 = Ystävä

4 = Muu, kuka?

Onko mielestäsi jokin tietyntyyppinen terveystieto erityisen arkaluontoista?

1 = Ei

2 = Kyllä, minkälainen?

Kuinka hyödyllisinä pitäisit seuraavia toimintoja, jos ne olisivat tarjolla?

1 = Täysin hyödytön

2 = Melko hyödytön

3 = Ei hyödytön eikä hyödyllinen

4 = Melko hyödyllinen

5 = Erittäin hyödyllinen

6 = En osaa sanoa

Yhteydenotto hoitohenkilökuntaan sähköisesti

Terveystiedoistani löytämieni virheiden ilmoittaminen

Kommenttien lisääminen terveystietoihini

Terveystietojen ilmoittaminen, esimerkiksi täyttämällä lomakkeen seuraavaa tapaamista varten

Mahdollisuus antaa tietoa omista odotuksista tulevaa käyntiä varten

Alue, jolla asut (sairaanhoitopiiri)

1 = Etelä-Karjala

2 = Etelä-Pohjanmaa

3 = Etelä-Savo

4 = Helsinki ja Uusimaa

5 = Itä-Savo

6 = Kainuu

7 = Kanta-Häme

8 = Keski-Pohjanmaa

9 = Keski-Suomi

10 = Kymenlaakso

11 = Lappi

12 = Länsi-Pohja

Sukupuolesi

1 = Nainen

2 = Mies

3 = Muu / En halua kertoa

Ylin koulutusasteesi

1 = Ei suoritettua koulutusta

2 = Perusaste (Peruskoulu tai kansakoulu)

3 = Ammattitutkinto/ylioppilas

4 = Opistoaste

5 = Alempi korkeakoulututkinto

6 = Ylempi korkeakoulututkinto

7 = Tohtorin tutkinto

8 = Jokin muu

Oletko terveydenhuollon ammattihenkilö?

1 = Kyllä

2 = Ei

Mikä seuraavista kuvaa parhaiten työtilannettasi? Valitse parhaiten sopiva vaihtoehto

1 = Kokoaikainen

2 = Osa-aikainen

3 = Opiskelija

4 = Eläkkeellä

5 = Työtön

6 = En pysty työskentelemään

7 = Ei mikään edellä mainituista

Finland

Swedish

Aalto-universitetet

Kanta

Användarenkät om Mina Kanta-sidor

Ålder

- ◯ 14 år eller under
- ◯ 15–17 år
- ◯ 18–19 år
- ◯ 20 – 24 år
- ◯ 25–34 år
- ◯ 35–44 år
- ◯ 45–54 år
- ◯ 55–64 år
- ◯ 65–74 år
- ◯ 75–84 år
- ◯ 85 år eller mer

Bedöm tjänsten Mina Kanta-sidor utgående från din erfarenhet (1 = Helt av annan åsikt, 7 = Helt av samma åsikt)

	1	2	3	4	5	6	7	Jag vet inte
Mina Kanta-sidors egenskaper motsvarar mina behov.	◯	◯	◯	◯	◯	◯	◯	◯

	1	2	3	4	5	6	7	Jag vet inte
Det är frustrerande att använda Mina Kanta-sidor.	◯	◯	◯	◯	◯	◯	◯	◯
Det är lätt att använda Mina Kanta-sidor.	◯	◯	◯	◯	◯	◯	◯	◯

Har du någon särskilt positiv erfarenhet av Mina Kanta-sidor?

◯ Ja

◯ Nej

Beskriv din positiva erfarenhet här så tydligt som möjligt:

____

____

____

Har du någon särskilt negativ erfarenhet av Mina Kanta-sidor?

◯ Ja

◯ Nej

Beskriv din negativa erfarenhet här så tydligt som möjligt:

____

____

____

Följande frågor gäller anteckningarna om din vård, dvs. undersökningsresultat och läkarens eller den övriga vårdpersonalens anteckningar om vårdbesök.

Hur ofta under de senaste 12 månaderna har du läst dina vårdanteckningar på Mina Kanta-sidor? (Välj det lämpligaste alternativet)

- Inte en enda gång
- Det här är första gången
- 2–9 gånger
- 10–20 gånger
- Över 20 gånger

Har någon av följande uppmanat dig att läsa vårdanteckningarna på Mina Kanta-sidor? (Välja alla alternativ som stämmer)

- [ ] Läkare
- [ ] Skötare
- [ ] Psykolog
- [ ] Fysioterapeut
- [ ] Annan vårdpersonal
- [ ] Skriftlig anvisning från hälso- och sjukvården
- [ ] Familj eller vänner
- [ ] Andra patienter
- [ ] Kanta.fi eller en annan officiell hälsowebbplats
- [ ] Tidningar, radio, tv eller sociala medier
- [ ] Annat, vad? ____________
- [ ] Ingen av dessa

Varför läser du anteckningarna om din vård på Mina Kanta-sidor? Ta ställning till följande påståenden.

	Helt av annan åsikt	Av relativt annan åsikt	Varken av samma eller av annan åsikt	Av relativt samma åsikt	Helt av samma åsikt
Av eget intresse	○	○	○	○	○
För att bättre förstå mitt hälsotillstånd	○	○	○	○	○
För att förbereda mig inför ett läkarbesök eller vård på sjukhus	○	○	○	○	○

	Helt av annan åsikt	Av relativt annan åsikt	Varken av samma eller av annan åsikt	Av relativt samma åsikt	Helt av samma åsikt
För att se min vårdhistoria	◯	◯	◯	◯	◯
För att försäkra mig om att jag förstått vad läkaren sagt	◯	◯	◯	◯	◯
För att följa de vårdföreskrifter jag fått	◯	◯	◯	◯	◯
Eftersom jag misstänker fel eller brister i uppgifterna	◯	◯	◯	◯	◯
För att dela informationen med släktingar	◯	◯	◯	◯	◯
För att dela informationen med vänner	◯	◯	◯	◯	◯
För att dela informationen med andra	◯	◯	◯	◯	◯
yrkesutbildade eller myndigheter som inte har tillgång till dem	◯	◯	◯	◯	◯
För att jag är osäker på om jag fått rätt vård	◯	◯	◯	◯	◯

Om du vill kan du uppge en annan anledning till att du läser vårdanteckningarna:

____

____

____

Vad tycker du om att du kan se anteckningarna om din vård om du vill? Ta ställning till följande påståenden.

	Helt av annan åsikt	Av relativt annan åsikt	Varken av samma eller av annan åsikt	Av relativt samma åsikt	Helt av samma åsikt
Åtkomsten stärker mitt förtroende för hälso- och sjukvården	◯	◯	◯	◯	◯
Åtkomsten stöder växelverkan mellan mig och hälso- och sjukvårdspersonalen	◯	◯	◯	◯	◯

Har du någon gång upptäckt ett fel i dina vårdanteckningar (om stav- och tryckfel inte beaktas)?

- ◯ Ja
- ◯ Nej
- ◯ Jag vet inte/jag minns inte

Hur allvarligt var det största felet du upptäckte?

- ◯ Inte alls allvarligt
- ◯ Rätt allvarligt
- ◯ Mycket allvarligt
- ◯ Jag är inte säker

Beskriv nedan det allvarligaste felet du upptäckt. (Lämna bort namn, andra personuppgifter och känslig information.)

Vi rapporterar inte detta (eller dina andra svar) till vården. Om felet gör dig orolig ska du diskutera det med din vårdinstans.

____

____

____

Har du någon gång upptäckt att det saknats information i dina vårdanteckningar?

- ◯ Ja
- ◯ Nej
- ◯ Jag vet inte/jag minns inte

Hur allvarlig var den största informationsbristen?

- ◯ Inte alls allvarlig
- ◯ Rätt allvarlig

- ◯ Mycket allvarlig
- ◯ Jag är inte säker

Beskriv nedan den allvarligaste bristen du upptäckt i informationen. (Lämna bort
namn, andra personuppgifter och känslig information.)

Vi rapporterar inte detta (eller dina andra svar) till vården. Om felet gör dig orolig ska du diskutera det med din vårdinstans.

____

____

____

Gjorde du något av följande när du upptäckte felet eller bristen i informationen?

- ◯ Jag informerade vårdpersonalen vid följande besök
- ◯ Jag kontaktade vården per telefon
- ◯ Jag gjorde ingenting
- ◯ Något annat, vad? ______________________________

Hur lätt är det för dig att upptäcka fel eller brister i dina vårdanteckningar?

- ◯ Mycket svårt
- ◯ Svårt
- ◯ Varken svårt eller enkelt
- ◯ Enkelt
- ◯ Mycket enkelt

Har du känt dig kränkt av något du läst på Mina Kanta-sidor?

- ◯ Nej
- ◯ Ja, hur? ______________________________

Följande frågor gäller din hälsa, vården och vårdanteckningarna.

Hur bedömer du din hälsa för närvarande?

◯ Mycket god

◯ Bra

◯ Ganska god

◯ Dålig

◯ Mycket dålig

◯ Jag vet inte eller vill inte uppge

Har du behandlats av en läkare eller annan hälso- och sjukvårdspersonal under
de senaste två (2) åren? (Välja alla alternativ som stämmer.)

☐ Ja, för min psykiska hälsa

☐ Ja, för cancersjukdomar

☐ Ja, för andra hälsoproblem

☐ Jag har inte fått behandling

Har du läst informationen om vården för din psykiska hälsa på Mina Kanta-sidorna?

◯ Ja, jag har läst all eller nästan all information

◯ Ja, jag har läst informationen delvis

◯ Jag har inte läst informationen

Har du läst informationen om din vård för cancer på Mina Kanta-sidorna?

◯ Ja, jag har läst all eller nästan all information

◯ Ja, jag har delvis läst informationen

◯ Jag har inte läst informationen

Var har du fått vård för din psykiska hälsa? (Välj alla alternativ som stämmer)

- [ ] Hälsovårdscentralen eller någon annan kommunal eller privat behandlingsenhet (t.ex. psykoterapi)
- [ ] Sjukhusets öppenvård (konsultation med specialist)
- [ ] Avdelningsvård på sjukhus
- [ ] Sjukhusjour

Hur länge har du fått vård för din psykiska hälsa?

- ◯ Mindre än 3 månader
- ◯ 3–12 månader
- ◯ 1–3 år
- ◯ Mer än 3 år

Följande frågor handlar om de multidisciplinära team som ibland engageras för att behandla sjukdomar. De består av experter från olika discipliner beroende på vilken sjukdom det handlar om.

Har din vård diskuterats på ett multidisciplinärt teammöte?

- ◯ Ja
- ◯ Nej
- ◯ Vet inte

Var du inbjuden till det multidisciplinära teamets möte när din vård diskuterades?

- ◯ Ja
- ◯ Nej

Hur upplevde du det multidisciplinära teamet?

Har du tillgång till promemorior från det multidisciplinära teamets möten?

- ◯ Ja
- ◯ Nej
- ◯ Jag vet inte

Följande frågor handlar om dina synpunkter på informationssäkerhet och integritet.

I vilken mån håller du med om följande påståenden?

	Helt av annan åsikt	Av relativt annan åsikt	Varken av samma eller av annan åsikt	Av relativt samma åsikt	Helt av samma åsikt
Jag litar på att endast befullmäktigad vårdpersonal läser min hälsoinformation	◯	◯	◯	◯	◯
Jag vill kunna ställa in vem som har tillgång till min hälsoinformation (t.ex. uppgifter om ett visst besök visas inte)	◯	◯	◯	◯	◯
Jag anser att Mina Kanta-sidor är informationssäkra	◯	◯	◯	◯	◯
Jag litar på att det är tryggt att logga in på Mina Kanta-sidor	◯	◯	◯	◯	◯

Har du kopierat dina uppgifter från Mina Kanta-sidorna till en webbapplikation t.ex. Google Health eller Facebook?

- ◯ Jag har aldrig kopierat information
- ◯ Jag har kopierat information några gånger (1–4 ggr.)
- ◯ Jag har ofta kopierat information (över 5 ggr.)

Har du upplevt att någon krävt att få se anteckningar om din vård som du inte hade velat dela?

- ◯ Ja
- ◯ Nej
- ◯ Vet inte

Vem har krävt att få se anteckningar om din vård som du inte hade velat dela?

- ◯ Familjemedlem
- ◯ Vän
- ◯ Någon annan, vem? ____________________

Har du upplevt att någon har sett anteckningar om din vård som du inte hade velat dela?

- ◯ Ja
- ◯ Nej
- ◯ Vet inte

Vem har sett anteckningar om din vård som du inte hade velat dela?

- ◯ Hälso-och sjukvårdspersonal
- ◯ Familjemedlem
- ◯ Vän
- ◯ Någon annan, vem? ____________________

Anser du att vissa typer av hälsoinformation är särskilt känsliga?

- ◯ Nej
- ◯ Ja, vilka? ____________________

Följande frågor handlar om e-tjänster som för närvarande inte finns på Mina Kanta-sidor

Hur användbara skulle följande funktioner vara enligt dig, om de skulle erbjudas?

	Helt onödig	Ganska onödig	Varken onödig eller användbar	Ganska användbar	Mycket användbar	Jag vet inte
Elektronisk kontaktkanal till vårdpersonalen	◯	◯	◯	◯	◯	◯
Rapportering av fel i min hälsoinformation	◯	◯	◯	◯	◯	◯
Möjlighet att kommentera min hälsoinformation	◯	◯	◯	◯	◯	◯
Rapportering av hälsoinformation, t.ex. genom att fylla i formulär inför följande besök	◯	◯	◯	◯	◯	◯
Möjlighet att lämna information om mina förväntningar på följande besök	◯	◯	◯	◯	◯	◯

Bakgrundsuppgifter

Område där du bor (sjukvårdsdistrikt)

- ◯ Södra Karelen
- ◯ Södra Österbotten
- ◯ Södra Savolax
- ◯ Helsingfors och Nyland
- ◯ Östra Savolax
- ◯ Kajanaland
- ◯ Egentliga Tavastland
- ◯ Mellersta Österbotten
- ◯ Mellersta Finland
- ◯ Kymmenedalen
- ◯ Lappland

- ◯ Länsi-Pohja
- ◯ Birkaland
- ◯ Norra Karelen
- ◯ Norra Österbotten
- ◯ Norra Savolax
- ◯ Päijänne-Tavastland
- ◯ Satakunta
- ◯ Vasa
- ◯ Egentliga Finland
- ◯ Åland
- ◯ Jag vet inte eller vill inte uppge

Kön

- ◯ Kvinna
- ◯ Man
- ◯ Annat/Vill inte uppge

Din högsta utbildningsnivå

- ◯ Ingen genomgången utbildning
- ◯ Grundnivå (Grundskola eller folkskola)
- ◯ Yrkesexamen/student
- ◯ Institutsnivå
- ◯ Lägre högskoleexamen
- ◯ Högre högskoleexamen
- ◯ Doktorsexamen
- ◯ Annan utbildning

Är du yrkesutbildad inom hälso- och sjukvården?

- ◯ Ja
- ◯ Nej

Vilket av följande beskriver bäst din arbetssituation? Välj det lämpligaste alternativet.

- ◯ Heltidsarbetande
- ◯ Deltidsarbetande
- ◯ Studerande
- ◯ Pensionerad
- ◯ Arbetslös
- ◯ Jag kan inte arbeta
- ◯ Inget av alternativen ovan

Estonia

Estonian

Survey

[Language]

Millises keeles eelistad vastata?

На каком языке Вы предпочитаете отвечать?

Row:

[r1] Eesti / На эстонском

[r2] Vene / На русском

Infokiri osalejatele

Täname Teid, et olete valmis osalema NORDeHEALTH teadusuuringus. Allolevast tekstist leiate lisainformatsiooni käesoleva projekti ja selles osalemise kohta.

Mis projektiga on tegu ja miks on minu osalus vajalik?

NORDeHEALTH on teadusprojekt, mille eesmärk on uurida inimeste kogemust patsiendiportaaliga digilugu.ee laiemalt ja seda milliseks hinnatakse juurdepääsu terviseandmetele. Teie kogemused on oluliseks sisendiks patsiendiportaali edasiseks arendamiseks. Lisaks Eestile viiakse uuring läbi Soomes, Rootsis ja Norras. Eestis on uuringu läbiviijaks Tallinna Tehnikaülikooli E-meditsiini keskus. Uuring on heaks kiidetud kõikide riikide eetikakomiteedes, Eestis on uuring saanud kooskõlastuse TAI inimuuringute eetikakomiteelt (otsus nr: 977).

Küsimustiku üldinfo

Osalemine võtab aega ligikaudu 10 minutit ja küsimustik tuleb täita järjest vahepeal vastamist katkestamata. Küsimustik koosneb 26 küsimusest, mis on seotud digilugu.ee e-teenuste kasutamisega.

Küsimustiku esitamisega kinnitate oma nõusoleku uuringus osalemiseks ja esitatud vastuste töötlemiseks.

Uuringus osalemine on vabatahtlik ning Te saate igal ajal osalemisest loobuda sulgedes brauseri akna, Te ei pea oma loobumisotsust kuidagi põhjendama.

Küsitlusega kogutavad andmed ja andmetöötlus

Kogutav teave on: peamist arstiabi osutav maakond, sugu, vanus, haridustase, ning küsimused selle kohta, kas olete saanud abi vaimse tervise valdkonnas või vähiraviga seoses. Kõik vastused on anonüümsed ja neid ei ole võimalik Teiega seostada.

Kogutud teabe eest vastutab Tallinna Tehnikaülikooli E-meditsiini keskus. Kui soovite lisainfot uuringu kohta, siis võtke ühendust Barbara Haage'ga, e-mail: barbara.haage@taltech.ee.

Uuringu ankeedi on programmeerinud Norstat Eesti AS, mis järgib vastuste kogumisel ja säilitamisel rahvusvahelise uuringuorganisatsiooni ESOMAR reegleid ning Euroopa Liidu andmekaitse direktiivi (GDPR) nõudeid. Vastuste kogumisel lähtutakse järgmistest privaatsuspõhimõtetest.

Kuidas saada teavet projekti tulemuste kohta?

Tulemustega on võimalik tutvuda pärast esialgse raporti valmimist (ligikaudne valmimisaeg on suvi 2022). Uuringutulemuste põhjal valmivad teadusartiklid ning ettekanded rahvusvahelistel konverentsidel. Projektiga on võimalus lähemalt tutvuda NORDeHEALTH veebisaidil https://nordehealth.eu/.

Hüvitis

Uuringus osalemise eest ei maksta hüvitist.

Osalemine on vabatahtlik.

[Q23] Vanus:

Row:

[r1] Alla 15 aasta

[r2] 15-24 aastat

[r3] 25-34 aastat

[r4] 35-44 aastat

[r5] 45-54 aastat

[r6] 55-64 aastat

[r7] 65-74 aastat

[r8] 75-84 aastat

[r9] 85 või rohkem aastat

Terminate: Screening: Q23.r1

Condition: **Q23.r1**

[Q1] Järgmised küsimused puudutavad Sinu seniseid kogemusi, mis on seotud oma terviseandmetele juurdepääsu ja nende lugemisega.

Palun hinda oma digiloo teenuse kasutamise kogemust skaalal 1-8 (1 - ei nõustu üldse; 7 - nõustun täielikult; 8 - ei tea).

Column:

[c1] 1 Ei nõustu üldse

[c2] 2

[c3] 3

[c4] 4

[c5] 5

[c6] 6

[c7] 7 Nõustun täielikult

[c8] 8 Ei tea

Row:

[r1] Digilugu vastab minu vajadustele

[r2] Digiloo kasutamine tekitab minus frustratsiooni

[r3] Digilugu on lihtne kasutada

[Q2] Kas Sul on olnud digiloo kasutamisel väga positiivset kogemust?

Row:

[r1] Jah

[r2] Ei

[Q2a] Palun kirjelda seda kogemust nii täpselt kui võimalik.

Condition: **Q2.r1**

[Q3] Kas Sul on olnud digiloo kasutamisel väga negatiivset kogemust?

Row:

[r1] Jah

[r2] Ei

[Q3a] Palun kirjelda seda kogemust nii täpselt kui võimalik.

Condition: **Q3.r1**

Järgmised küsimused puudutavad Sinu seniseid kogemusi, mis on seotud oma terviseandmetele juurdepääsu ja nende lugemisega.

[Q4] Mitu korda Sa arvad, et oled vaadanud oma terviseandmeid viimase 12 kuu jooksul?

Row:

[r1] Praegu on esimene kord

[r2] 2-9 korda

[r3] 10-20 korda

[r4] Rohkem kui 20 korda

[r5] Ma ei ole kunagi vaadanud oma terviseandmeid

[Q5] Mis või kes ajendas Sind või tuletas Sulle meelde oma terviseandmetega digiloos tutvuma? Märgi sobivad valikud, vajadusel rohkem kui üks.

Condition: **Q4.any and not Q4.r5**

Row:

[r1] Arst

[r2] Õde

[r3] Psühholoog

[r4] Füsioterapeut

[r5] Muu meditsiiniline personal

[r6] Tervishoiuspetsialist – ei mäleta

Condition: **0**

[r7] Kirjalik informatsioon haiglas ja/või tervishoiuasutuses

[r8] Perekond või sõbrad

[r9] Internetilehekülg, näiteks digilugu.ee

[r10] Ajalehed, raadio, TV, sotsiaalmeedia vms

[r11] Mõni teine patsient

[r12] Muu

[r13] Miski/keegi ei ajendanud mind

[Q6] Palun märgi, mil määral Sa ei nõustu või nõustud järgmiste väidetega skaalal 1-5.

Condition: **Q4.any and not Q4.r5**

1 - ei nõustu üldse; 2 - ei nõustu; 3 - neutraalne, 4 - nõustun, 5 - nõustun täielikult.

Ma vaatasin oma terviseandmeid digiloost:

Column:

[c1] 1 Ei nõustu üldse

[c2] 2

[c3] 3 Nii ja naa

[c4] 4

[c5] 5 Nõustun täielikult

Row:

[r1] Uudishimust

[r2] Selleks, et saada paremini aru oma tervisemurest

[r3] Selleks, et valmistuda visiidiks või haiglaraviks

[r4] Selleks, et saada ülevaade oma haigusloost

[r5] Selleks, et kontrollida, kas sain arsti juhistest õigesti aru

[r6] Selleks, et järgida oma ravisoovitusi

[r7] Sest kahtlustan sisestatud andmetes ebatäpsusi

[r8] Selleks, et jagada oma terviseandmeid lähisugulastega

[r9] Selleks, et jagada oma terviseandmeid sõpradega

[r10] Selleks, et jagada enda terviseandmeid tervishoiuteenuste osutajatega, kellel puudub ligipääs minu andmetele

[r11] Sest ma ei ole kindel, kas sain õiget ravi

[r12] Muu (palun täpsusta):

[Q7] Palun märgi, mil määral Sa ei nõustu või nõustud järgmiste väidetega skaalal 1-5.

Condition: **Q4.any and not Q4.r5**

(1 - ei nõustu üldse; 2 - ei nõustu; 3 - neutraalne, 4 - nõustun,5 - nõustun täielikult).

Ligipääs enda terviseandmetele:

Column:

[c1] 1 Ei nõustu üldse

[c2] 2

[c3] 3 Nii ja naa

[c4] 4

[c5] 5 Nõustun täielikult

Row:

[r1] Aitab mul usaldada oma tervishoiuteenuse osutajat

[r2] Toetab paremat suhtlust minu ja tervishoiutöötajate vahel

---

[Q8] Kas oled oma digiloo andmetes lugenud midagi, mis Sinu arvates on valesti kirja pandud (v.a. kirjavead)?

Condition: **Q4.any and not Q4.r5**

Row:

[r1] Jah

[r2] Ei

[r3] Ei tea / ei mäleta

[Q8a] Kui tõsine oli kõige suurem viga Sinu jaoks?

Condition: **Q8.r1**

Row:

[r1] Ei olnud üldse tõsine

[r2] Mõnevõrra tõsine

[r3] Väga tõsine

[r4] Ma ei ole kindel

[Q8b] Palun kirjelda kõige tõsisemat viga (ära maini nimesid, isikutunnuseid või muud delikaatset informatsiooni).

Condition: **Q8.r1**

NB! Me ei edasta seda (ega ka mingit muud informatsiooni selles küsimustikus). Sinu tervishoiuteenuse osutajale. Kui oled mures võimaliku vea pärast, siis palun võta ühendust vastava tervishoiuteenuse osutajaga.

[Q9] Kas oled oma digiloo andmetes lugenud midagi, mis Sinu arvates on puudu (v.a. kirjavead)?

Condition: **Q4.any and not Q4.r5**

Row:

[r1] Jah

[r2] Ei

[r3] Ei tea / ei mäleta

[Q9a] Kui tõsine oli kõige olulisem puuduolev informatsioon Sinu jaoks?

Condition: **Q9.r1**

Row:

[r1] Ei olnud üldse tõsine

[r2] Mõnevõrra tõsine

[r3] Väga tõsine

[r4] Ma ei ole kindel

[Q9b] Palun kirjelda kõige tõsisemat puuduolevat informatsiooni (ära maini nimesid, isikutunnuseid või muud delikaatset informatsiooni).

Condition: **Q9.r1**

NB! Me ei edasta seda (ega ka mingit muud informatsiooni selles küsimustikus). Sinu tervishoiuteenuse osutajale. Kui oled mures võimaliku vea pärast, siis palun võta ühendust vastava tervishoiuteenuse osutajaga.

[Q9c] Kui avastasid vea või puuduoleva informatsiooni oma terviseandmetes, siis kas tegid midagi alljärgnevatest (vali kõige olulisem)?

Condition: **Q8.r1 or Q9.r1**

Row:

[r1] Teavitasin sellest järgmisel visiidil tervishoiutöötajat

[r2] Võtsin telefoni teel ühendust tervishoiuteenuse osutajaga

[r3] Ma ei teinud midagi

[r4] Muu (palun täpsusta)

[Q10] Kui keeruline või lihtne on Sinul leida vigu oma terviseandmetes skaalal 1-5 (1 - väga keeruline; 2 - keeruline; 3 - neutraalne, 4 - lihtne, 5 - väga lihtne)?

Condition: **Q4.any and not Q4.r5**

Column:

[c1] 1 Väga keeruline

[c2] 2

[c3] 3 Ei keeruline ega lihtne

[c4] 4

[c5] 5 Väga lihtne

[Q11] Kas oled kunagi tundnud end solvatuna millestki, mida oled lugenud oma terviseandmetes?

Condition: **Q4.any and not Q4.r5**

Row:

[r1] Jah

[r2] Ei

[Q11a] Palun täpsusta, mille tõttu tundsid end solvatuna?

Condition: **Q11.r1**

Järgmised küsimused puudutavad Sinu tervist, saadud ravi ja selle dokumenteeritud andmeid.

[Q12] Kuidas hindad enda tervislikku seisundit?

Row:

[r1] Väga hea

[r2] Hea

[r3] Rahuldav

[r4] Halb

[r5] Väga halb

[r6] Ei oska öelda/ei soovi vastata

[Q13] Kas Sa oled viimase kahe aasta jooksul saanud arstilt (perearstilt või eriarstilt) meditsiinilist abi?

Märgi sobivad valikud - vajadusel rohkem kui üks.

Row:

[r1] Vaimse tervisega seoses

[r2] Vähiraviga seoses

[r3] Muu terviseprobleemiga seoses

[r4] Ei ole saanud meditsiinilist abi

[Q13a1] Kas oled lugenud digiloos enda vaimse tervise raviga seotud andmeid?

Condition: **Q13.r1**

Row:

[r1] Ma olen lugenud sellega seonduvaid raviandmeid täies mahus/peaaegu täies mahus

[r2] Ma olen lugenud ainult vähese osa sellega seonduvaid raviandmeid

[r3] Ma ei ole lugenud enda raviandmeid

[Q13a2] Kas oled lugenud digiloos enda vähiraviga seotud andmeid?

Condition: **Q13.r2**

Row:

[r1] Ma olen lugenud sellega seonduvaid raviandmeid täies mahus/peaaegu täies mahus

[r2] Ma olen lugenud ainult vähese osa sellega seonduvaid raviandmeid

[r3] Ma ei ole lugenud enda raviandmeid

[Q13b] Millist abi oled saanud vaimse tervisega seoses (märgi sobivad valikud – vajadusel rohkem kui üks)?

Condition: **Q13.r1**

Row:

[r1] Esmatasand (näiteks perearst)

[r2] Ambulatoorne eriarstiabi (haiglaväline ravi, päevaravi)

[r3] Statsionaarne eriarstiabi (haiglaravi)

[r4] Erakorraline abi (EMO)

[Q13c] Kui kaua oled saanud ravi seoses vaimse tervisega?

Condition: **Q13.r1**

Row:

[r1] Vähem kui 3 kuud

[r2] 3 kuud kuni 1 aasta

[r3] 1-3 aastat

[r4] Üle 3 aasta

[Q14] Kas Sinu raviplaani on arutatud meditsiinilisel konsiiliumil?

Meditsiiniline konsiilium - erinevate erialade arstidest koosneva spetsialistide grupi kohtumine, mille käigus otsustatakse keerulisemate haigete raviplaan.

Row:

[r1] Jah

[r2] Ei

[r3] Ei oska öelda

[Q14a] Kas Sind kutsuti konsiiliumi kohtumisele?

Condition: **Q14.r1**

Row:

[r1] Jah

[r2] Ei

[Q14b] Milline on olnud Sinu kogemus konsiiliumi(te)ga?

Condition: **Q14.r1**

[Q14c] Kas Sul on digiloos ligipääs konsiiliumi dokumentidele?

Condition: **Q14.r1**

Row:

[r1] Jah

[r2] Ei

[r3] Ei tea

[Q15] Mis on Sinu arvamus digiloo turvalisuse ja privaatsuse kohta?

Palun märgi, kui palju Sa ei nõustu või nõustud järgmiste väidetega skaalal 1-5 (1 - ei nõustu üldse; 2 - ei nõustu; 3 - neutraalne, 4 - nõustun, 5 - nõustun täielikult).

Column:

[c1] 1 Ei nõustu üldse

[c2] 2

[c3] 3 Nii ja naa

[c4] 4

[c5] 5 Nõustun täielikult

Row:

[r1] Minu hinnangul on digiloo kasutamine väga turvaline

[r2] Usun, et minu terviseandmetele pääsevad ligi ainult volitatud tervishoiutöötajad

[r3] Ma usaldan digiloo sisselogimisprotsessi

[r4] Ma ei näe privaatsusprobleemi enda kogutud terviseandmete sisestamisel digilukku (nt ülevaade treeningutest, EKG tulemused või terviseandmed nutiseadmetest)

[r5] Ma ei näe privaatsusprobleemi oma terviseandmete kopeerimisel digiloost teistesse rakendustesse (nt. Google Health, Apple Health, Facebook jne)

[r6] Ma tahan, et mul oleks võimalik hallata, kellel on ligipääs minu terviseandmetele (nt. mingi kindel diagnoos ei ole ligipääsetav teatud tervishoiuteenuse osutajatele või sugulastele)

[Q16] Kui tihti kopeerid ja kleebid oma terviseandmeid digiloost teistesse rakendustesse (nt. Google Health,

Apple Health, Facebook jne)?

Row:

[r1] Ma ei ole seda teinud

[r2] Olen seda teinud mõned korrad (1-4 korda)

[r3] Ma teen seda tihti (rohkem kui 5 korda)

[Q17] Kas oled kogenud, et perekonnaliikmed, sõbrad või keegi teine on nõudnud ligipääsu Sinu andmetele, mida Sina ei ole tahtnud jagada?

Row:

[r1] Jah

[r2] Ei

[r3] Ei oska öelda

[Q17a] Kes nõudis ligipääsu Sinu terviseandmetele?

Condition: **Q17.r1**

Row:

[r1] Perekonnaliige

[r2] Sõber

[r3] Muu (palun täpsusta):

[Q18] Kas oled kogenud, et keegi on näinud Sinu terviseandmeid, mida Sina ei ole tahtnud jagada?

Row:

[r1] Jah

[r2] Ei

[r3] Ei oska öelda

[Q18a] Kes nägi Sinu terviseandmeid, mida Sina ei

tahtnud jagada?

Condition: **Q18.r1**

Row:

[r1] Tervishoiutöötaja

[r2] Perekonnaliige

[r3] Sõber

[r4] Muu (palun täpsusta):

[Q19] Kas teatud tüüpi terviseandmed on Sinu hinnangul eriti tundliku sisuga?

Row:

[r1] Jah

[r2] Ei

[Q19a] Kas oskad välja tuua, millised terviseandmed on Sinu jaoks eriti tundliku sisuga?

Condition: **Q19.r1**

Järgmised küsimused puudutavad Sinu arvamust digiloo uute funktsioonide ja võimaluste kohta.

[Q20] Järgnevalt on nimetatud dokumendid ja informatsioon, mis hetkel ei ole digiloos kättesaadavad. Millistele nendest oleks Sul kasulik ligipääsu omada (märgi sobivad valikud - vajadusel rohkem kui üks)?

Row:

[r1] Võimalus lugeda kogu infot enda tervise kohta, mida tervishoiutöötaja kogub

[r2] Ülevaade kõigist enda raviga seotud tervishoiutöötajatest ja nende kontaktid

---

[Q20a] Järgnevalt on nimetatud funktsioonid, mis hetkel ei ole digiloos kättesaadavad. Millistele nendest oleks Sul kasulik ligipääsu omada (märgi sobivad valikud - vajadusel rohkem kui üks)?

Row:

[r1] Võimalus blokeerida teatud tervishoiutöötajatele ligipääs osadele terviseandmetele.

[r2] Võimalus võtta oma tervishoiuteenuse osutajaga (nt perearst või haigla) ühendust interneti teel ja küsida küsimusi oma terviseandmete kohta.

[r3] Võimalus märgistada oma terviseandmetest leitud vead.

[r4] Võimalus lisada oma epikriisidesse (ravi kokkuvõte) kommentaare.

[r5] Võimalus taotleda haiguslehte.

[r6] Võimalus lisada enda terviseandmeid (nt. kodused vererõhu mõõtmised, veresuhkru mõõtmised jne).

[r7] Võimalus lisada infot enne vastuvõttu.

Järgmised küsimused puudutavad Sinu tausta.

[Q21] Maakond, kus elad:

Row:

[r1] Harju maakond

[r2] Hiiumaa

[r3] Ida-Virumaa

[r4] Jõgevamaa

[r5] Järva maakond

[r6] Läänemaa

[r7] Lääne-Virumaa

[r8] Põlvamaa

[r9] Pärnu maakond

[r10] Raplamaa

[r11] Saaremaa

[r12] Tartumaa

[r13] Valgamaa

[r14] Viljandimaa

[r15] Võrumaa

[Q22] Sugu:

Row:

[r1] Naine

[r2] Mees

[r3] Muu

[Q24] Haridustase:

Row:

[r1] Alghariduseta

[r2] Algharidus

[r3] Põhiharidus

[r4] Keskharidus

[r5] Kutseharidus

[r6] Bakalaureus või sellega võrdsustatud haridus

[r7] Magister või sellega võrdsustatud haridus

[r8] Doktor või sellega võrdsustatud haridus

[Q25] Kas Sa omad tervishoiualast kõrgharidust?

Row:

[r1] Jah

[r2] Ei

[Q26] Milline järgnevatest kirjeldab Sinu hetke tööstaatust kõige paremini?

Row:

[r1] Täistööaeg

[r2] Osaline tööaeg

[r3] Õpilane/üliõpilane

[r4] Pensionär

[r5] Töötu

[r6] Töövõimetu

[r7] Mitte ükski nimetatutest (kirjeldage):

COMPLETE - testing

Your responses will NOT be saved.

You may now close this window or use the "Back" button to keep testing the survey.

Estonia

Russian

Survey

[Language]

Millises keeles eelistad vastata?

На каком языке Вы предпочитаете отвечать?

Ряд:

[r1] Eesti / На эстонском

[r2] Vene / На русском

Информационное письмо для участников

Благодарим Вам за готовность принять участие в научном исследовании NORDeHEALTH Research. Этот текст содержит дополнительную информацию об этом проекте и участии в нем.

Что это за проект и почему необходимо мое участие?

NORDeHEALTH - это научный проект, целью которого является изучение опыта взаимодействия пользователей с порталом для пациентов digilugu.ee в целом и оценка доступа к данным о состоянии здоровья. Ваш опыт послужит важным вкладом в дальнейшее развитие портала для пациентов. Наряду с Эстонией, опрос проводится в Финляндии, Швеции и Норвегии. В Эстонии опрос проводится Центром электронной медицины Таллиннского технологического университета. Исследование было одобрено комитетами по этике всех стран, в Эстонии исследование было одобрено Комитетом по этике исследований на людях при Институте развития здоровья (решение №: 977).

Общая информация по анкете

Участие занимает около 15-20 минут, и анкета должна заполняться подряд и без перерыва. Анкета состоит из 26 вопросов, связанных с использованием электронных услуг digilugu.ee.

Отправляя анкету, вы подтверждаете свое согласие на участие в опросе и обработку полученных ответов.

Участие в опросе является добровольным, и Вы можете отказаться в любой момент, закрыв окно браузера, без необходимости объяснения причин своего решения об отказе.

Данные опросов и обработка данных

Собирается следующая информация: уезд, предоставляющий первичную медицинскую помощь, пол, возраст, уровень образования, а также вопросы о том, получали ли вы помощь с сфере психического здоровья или в связи с лечением рака. Все ответы анонимны и не могут быть связаны с Вами.

За собранную информацию отвечает центр электронной медицины Таллиннского технологического университета. Если вам нужна дополнительная информация об опросе, пожалуйста, свяжитесь с Барбарой Хааге, электронная почта: barbara.haage@taltech.ee.

Анкета для опроса была разработана Norstat Eesti AS с соблюдением правил сбора и хранении ответов международной исследовательской организации ESOMAR и требований Директивы Европейского Союза о защите данных (GDPR). Ответы будут собираться в соответствии со следующими политиками конфиденциальности.

Как получить информацию о результатах проекта?

Результаты будут доступны после завершения первоначального отчета (ориентировочная дата завершения - лето 2022 года). По результатам исследования будут подготовлены научные статьи и презентации на международных конференциях. Вы можете узнать больше о проекте на сайте NORDeHEALTH https://nordehealth.eu/.

Вознаграждение

Вознаграждение за участие в исследовании не выплачивается.

Участие добровольное.

[Q23] Возраст:

Ряд:

[r1] Менее 15 лет

[r2] 15-24 лет

[r3] 25-34 лет

[r4] 35-44 лет

[r5] 45-54 лет

[r6] 55-64 лет

[r7] 65-74 лет

[r8] 75-84 лет

[r9] 85 и более лет

Terminate: Screening: Q23.r1

Condition: **Q23.r1**

[Q1] Следующие вопросы касаются Вашего прежнего опыта, связанного с доступом к своим данным о здоровье и чтением этих данных.

Пожалуйстя, оцените свой опыт использования портала для пациентов по шкале от 1 до 8 (1 – совершенно не согласен; 7 – полностью согласен; 8 – не знаю).

Столбец:

[c1] 1 Совершенно не согласен

[c2] 2

[c3] 3

[c4] 4

[c5] 5

[c6] 6

[c7] 7 Полностью

[c8] 8 Не знаю

Ряд:

[r1] Портал для пациентов соответствует моим потребностям

[r2] Использование портала для пациентов вводит меня в замешательство

[r3] Портал для пациентов прост в использовании

[Q2] Был ли у Вас очень положительный опыт использования портала для пациентов?

Ряд:

[r1] Да

[r2] Нет

[Q2a] Пожалуйста, опишите этот опыт как можно точнее.

Condition: **Q2.r1**

[Q3] Был ли у Вас очень негативный опыт использования портала для пациентов?

Ряд:

[r1] Да

[r2] Нет

[Q3a] Пожалуйста, опишите этот опыт как можно точнее.

Condition: **Q3.r1**

Следующие вопросы касаются Вашего прежнего опыта, связанного с доступом к своим данным о здоровье и чтением этих данных.

[Q4] По Вашей оценке, сколько раз за последние 12 месяцев Вы просматривали свои данные о здоровье?

Ряд:

[r1] Сейчас впервые

[r2] 2–9 раз

[r3] 10-20 раз

[r4] Более 20 раз

[r5] Я никогда не смотрел свои данные о здоровье

[Q5] Что или кто побудил Вас или напомнил вам ознакомиться со своими данными о здоровье на портале для пациента? Отметьте подходящие варианты, если нужно, то больше одного.

Condition: **Q4.any and not Q4.r5**

Ряд:

[r1] Врач

[r2] Сестра

[r3] Психолог

[r4] Физиотерапевт

[r5] Другой медицинский персонал

[r6] Специалист сферы здравоохранения - не помню

Condition: **0**

[r7] Письменная информация в больнице и/или медицинском учреждении

[r8] Семья или друзья

[r9] Веб-сайт, например digilugu.ee

[r10] Газеты, радио, телевидение, социальные сети и т.д.

[r11] Другой пациент

[r12] Иное

[r13] Меня ничто/никто не побуждал

[Q6] Пожалуйста, укажите, в какой мере Вы не согласны или согласны со следующими утверждениями по шкале от 1 до 5

Condition: **Q4.any and not Q4.r5**

1 – совершенно не согласен; 2 – не согласен; 3 – отношусь нейтрально, 4 – согласен, 5 – полностью согласен

Я посмотрел данные о своем здоровье на портале для пациента:

Столбец:

[c1] 1 Совершенно не согласен

[c2] 2

[c3] 3 Нейтрально

[c4] 4

[c5] 5 Полностью согласен

Ряд:

[r1] Из любопытства

[r2] Чтобы лучше понимать свои проблемы со здоровьем

[r3] Чтобы подготовиться к визиту или лечению в больнице

[r4] Чтобы получить обзор своей истории болезни

[r5] Чтобы проверить, правильно ли я понял указания врача

[r6] Чтобы соблюдать свои рекомендации по лечению

[r7] Потому что я подозреваю неточности во введенных данных

[r8] Чтобы поделиться информацией о своем здоровье с близкими

[r9] Чтобы поделиться информацией о своем здоровье с друзьями

[r10] Чтобы поделиться информацией о своем здоровье с поставщиками услуг здравоохранения, у которых отсутствует доступ к моим данным

[r11] Потому что я не уверен, что получил правильное лечение

[r12] Другое (пожалуйста, уточните)...

[Q7] Пожалуйста, укажите, в какой степени Вы не согласны или согласны со следующими утверждениями по шкале от 1 до 5.

Condition: **Q4.any and not Q4.r5**

(1 – совершенно не согласен; 2 – не согласен; 3 – отношусь нейтрально, 4 – согласен, 5 – полностью согласен)

Столбец:

[c1] 1 Совершенно не согласен

[c2] 2

[c3] 3 Нейтрально

[c4] 4

[c5] 5 Полностью согласен

Ряд:

[r1] Помогает мне доверять своему поставщику услуг здравоохранения

[r2] Поддерживает лучшее общение между мной и работниками здравоохранения

[Q8] Вы читали у себя на портале для пациента что-то, что, по Вашему мнению, записано неверно (за исключением грамматических ошибок)?

Condition: **Q4.any and not Q4.r5**

Ряд:

[r1] Да

[r2] Нет

[r3] Не знаю / не помню

[Q8a] Насколько серьезной была самая большая ошибка для Вас?

Condition: **Q8.r1**

Ряд:

[r1] Совершенно несерьезная

[r2] В некоторой степени серьезная

[r3] Очень серьезная

[r4] Я не уверен

[Q8b] Пожалуйста, опишите самую серьезную ошибку (не упоминайте имена, идентификаторы личности или другую деликатную информацию).

Condition: **Q8.r1**

NB! Мы не будем передавать эту информацию (или любую другую информацию из этой анкеты). Вашему поставщику услуг здравоохранения. Если Вас беспокоит возможная ошибка, пожалуйста, обратитесь к соответствующему поставщику услуг здравоохранения.

[Q9] Вы читали у себя на портале для пациента что-то, что, по Вашему мнению, записано недостаточно (за исключением грамматических ошибок)?

Condition: **Q4.any and not Q4.r5**

Ряд:

[r1] Да

[r2] Нет

[r3] Не знаю / не помню

[Q9a] Насколько серьезной была для Вас самая важная недостающая информация?

Condition: **Q9.r1**

Ряд:

[r1] Совершенно несерьезная

[r2] В некоторой степени серьезная

[r3] Очень серьезная

[r4] Я не уверен

[Q9b] Пожалуйста, опишите самую серьезную недостающую информацию (не упоминайте имена, идентификаторы личности или другую деликатную информацию).

Condition: **Q9.r1**

NB! Мы не будем передавать эту информацию (или любую другую информацию из этой анкеты). Вашему поставщику услуг здравоохранения. Если Вас беспокоит возможная ошибка, пожалуйста, обратитесь к соответствующему поставщику услуг здравоохранения.

[Q9c] Когда Вы обнаружили ошибку или недостающую информацию в своих данных о

здоровье, сделали ли Вы что-либо из перечисленного ниже (выберите наиболее важное)?

Condition: **Q8.r1 or Q9.r1**

Ряд:

[r1] Я проинформировал об этом работника здравоохранения во время следующего визита

[r2] Я связался с поставщиком услуги здравоохранения по телефону

[r3] Я ничего не делал

[r4] Другое (пожалуйста, уточните)

[Q10] Насколько сложно или просто Вам найти ошибки в своих данных о здоровье по шкале от 1 до 5 (1 – очень сложно; 2 – сложно; 3 – нейтрально, 4 – просто, 5 – очень просто)?

Condition: **Q4.any and not Q4.r5**

Столбец:

[c1] 1 Очень сложно

[c2] 2

[c3] 3 Ни сложно, ни просто

[c4] 4

[c5] 5 Очень просто

[Q11] Вы когда-нибудь чувствовали себя оскорбленными чем-то, что прочитали в своих данных о здоровье?

Condition: **Q4.any and not Q4.r5**

Ряд:

[r1] Да

[r2] Нет

[Q11a] Пожалуйста, уточните, по какой причине Вы почувствовали себя оскорбленным?

Condition: **Q11.r1**

Следующие вопросы касаются Вашего здоровья, полученного лечения и задокументированных данных о нем.

[Q12] Как Вы оцениваете состояние своего здоровья?

Ряд:

[r1] Очень хорошее

[r2] Хорошее

[r3] Удовлетворительное

[r4] Плохое

[r5] Очень плохое

[r6] Затрудняюсь ответить / не хочу отвечать

[Q13] В течение последних двух лет Вы получали медицинскую помощь от врача (семейного врача или специалиста)?

Укажите подходящие варианты, при необходимости более одного.

Ряд:

[r1] В связи с душевным здоровьем

[r2] В связи с лечением от рака

[r3] В связи с другой проблемой со здоровьем

[r4] Не получал медицинской помощи

[Q13a1] Вы читали на портале пациента свои данные, связанные с лечением душевным здоровьем?

Condition: **Q13.r1**

Ряд:

[r1] Я в полном/почтиполном объеме прочитал связанные с этим данные о лечении.

[r2] Я прочитал только небольшую часть связанных с этим данных о лечении.

[r3] Я не читал данные о своем лечении.

[Q13a2] Вы читали на портале пациента свои данные, связанные с лечением от рака?

Condition: **Q13.r2**

Ряд:

[r1] Я в полном/почтиполном объеме прочитал связанные с этим данные о лечении.

[r2] Я прочитал только небольшую часть связанных с этим данных о лечении.

[r3] Я не читал данные о своем лечении.

[Q13b] Какую помощь Вы получили в связи с душевным здоровьем (отметьте подходящие варианты – при необходимости более одного)?

Condition: **Q13.r1**

Ряд:

[r1] Начальный уровень (например, семеыный врач)

[r2] Амбулаторное лечение у специалистов (лечение вне больницы, дневное лечение)

[r3] Стационарное лечение у специалистов (лечение в больнице)

[r4] Скорая помощь (EMO)

[Q13c] Если отмечено душевное здоровье, то: Как долго Вы получали лечение в связи с душевным здоровьем?

Condition: **Q13.r1**

Ряд:

[r1] Менее 3 месяцев

[r2] От 3 месяцев до 1 года

[r3] 1-3 года

[r4] Более 3 лет

[Q14] Обсуждали ли Ваш план лечения на медицинском консилиуме?

Медицинский консилиум – это собрание группы медицинских специалистов, состоящее из врачей разных специальностей, в ходе которого определяется план лечения для более сложных пациентов.

Ряд:

[r1] Да

[r2] Нет

[r3] Затрудняюсь ответить

[Q14a] Вас приглашали на консилиум?

Condition: **Q14.r1**

Ряд:

[r1] Да

[r2] Нет

[Q14b] Каков Ваш опыт с консилиумом(-ами)?

Condition: **Q14.r1**

[Q14c] Есть ли у Вас на портале для пациента доступ к документам консилиума?

Condition: **Q14.r1**

Ряд:

[r1] Да

[r2] Нет

[r3] Не знаю

[Q15] Что Вы думаете о безопасности и конфиденциальности данных на портале для пациентов?

Пожалуйста, укажите, в какой степени Вы не согласны или согласны со следующими утверждениями по шкале от 1 до 5 (1 – совершенно не согласен; 2 – не согласен; 3 – отношусь нейтрально, 4 – согласен, 5 – полностью согласен).

Столбец:

[c1] 1 Совершенно не согласен

[c2] 2

[c3] 3 Нейтрально

[c4] 4

[c5] 5 Полностью согласен

Ряд:

[r1] По моей оценке, использование портала для пациентов очень безопасно.

[r2] Я верю, что доступ к моим данным о здоровье имеют только уполномоченные работники сферы здравоохранения.

[r3] Я доверяю процессу входа в портал для пациента.

[r4] Я не вижу проблем с конфиденциальностью, когда ввожу данные о своем здоровье в портал для пациента (например, обзор тренировок, результаты ЭКГ или данные о здоровье со смарт-устройства).

[r5] Я не вижу проблем с конфиденциальностью при копировании данных о моем здоровье с портала для пациента в другие приложения (например, Google Health, Apple Health, Facebook и т.д.).

[r6] Я хочу, чтобы у меня была возможность управлять тем, кому давать доступ к данным о моем здоровье (например, чтобы определенный диагноз был недоступен для определенных поставщиков услуг здравоохранения или родственников)

[Q16]

Как часто Вы копируете данные о своем здоровье из портала для пациента в другие приложения (например, Google Health, Apple Health, Facebook и т.д.)?

Ряд:

[r1] Я этого не делал

[r2] Я делал это несколько раз (1-4 раза)

[r3] Я делаю это часто (более 5 раз)

[Q17] Сталкивались ли Вы с тем, что члены семьи, друзья или кто-то еще запрашивал доступ к Вашим данным, которыми Вы не хотели делиться?

Ряд:

[r1] Да

[r2] Нет

[r3] Затрудняюсь ответить

[Q17a] Кто запрашивал доступ к Вашим данным о здоровье?

Condition: **Q17.r1**

Ряд:

[r1] Член семьи

[r2] Друг

[r3] Другое (пожалуйста, уточните):

[Q18] Сталкивались ли Вы с тем, что кто-то видел данные о Вашем здоровье, которыми Вы не хотели делиться?

Ряд:

[r1] Да

[r2] Нет

[r3] Затрудняюсь ответить

[Q18a] Кто видел Ваши данные о здоровье, которыми Вы не хотели делиться?

Condition: **Q18.r1**

Ряд:

[r1] Работник сферы здравоохранения

[r2] Член семьи

[r3] Друг

[r4] Другое (пожалуйста, уточните):

[Q19]

Являются ли, по Вашей оценке, определенные типы данных о здоровье особенно деликатными?

Ряд:

[r1] Да

[r2] Нет

[Q19a]

Можете ли вы указать, какие данные о здоровье для вас особенно деликатны?

Condition: **Q19.r1**

Следующие вопросы касаются Вашего мнения о новых функциях и возможностях портала для пациента.

[Q20] Ниже приведены документы и информация, которые в настоящее время недоступны на портале

для пациента. К каким из них Вам было бы полезно иметь доступ (отметьте подходящие варианты, если нужно, то больше одного)?

Ряд:

[r1] Возможность прочитать всю информацию о своем здоровье, которую собирает медицинский работник.

[r2] Обзор всех работников здравоохранения, участвующих в моем лечении, и их контакты.

[Q20a] Ниже приведены функции, которые в настоящее время недоступны на портале для пациента. К каким из них Вам было бы полезно иметь доступ (отметьте подходящие варианты, если нужно, то больше одного)?

Ряд:

[r1] Возможность заблокировать для определенного работника здравоохранения доступ к некоторым данным о здоровье.

[r2] Возможность связаться по интернету со своим поставщиком услуги здравоохранения (например, семейный врач или больница) и задать вопросы по поводу своих данных о здоровье.

[r3] Возможность обозначить ошибки, найденные в своих данных о здоровье.

[r4] Возможность добавлять комментарии к своим эпикризам (краткое описание лечения).

[r5] Возможность отправить ходатайство о получении больничного листа.

[r6] Возможность добавлять данные о своем здоровье (например, домашние измерения кровяного давления, измерения содержания сахара в крови и т.д.).

[r7] Возможность добавить информацию перед приемом.

Следующие вопросы касаются фоновой информации о Вас

[Q21] Уезд, в котором Вы живете:

Ряд:

[r1] Харьюмаа

[r2] Хийумаа

[r3] Ида-Вирумаа

[r4] Йыгевамаа

[r5] Ярваский уезд

[r6] Ляэнемаа

[r7] Ляэне-Вирумаа

[r8] Пылвамаа

[r9] Пярнуский уезд

[r10] Рапламаа

[r11] Сааремаа

[r12] Тартумаа

[r13] Валгамаа

[r14] Вильяндимаа

[r15] Вырумаа

[Q22] Пол:

Ряд:

[r1] Женщина

[r2] Мужчина

[r3] Другой

[Q24] Уровень образования

Ряд:

[r1] Нет начального образования

[r2] Начальное образование

[r3] Основное образование

[r4] Среднее образование

[r5] Профессиональное образование

[r6] Степень бакалавра или приравниваемое к нему образование

[r7] Степень магистра или приравниваемое к нему образование

[r8] Степень доктора или приравниваемое к нему образование

[Q25] У Вас есть высшее образование в сфере здравоохранения?

Ряд:

[r1] Да

[r2] Нет

[Q26] Что из перечисленного ниже лучше всего описывает Ваш нынешний статус занятости?

Ряд:

[r1] Полная ставка

[r2] Неполная ставка

[r3] Учащийся/студент

[r4] Пенсионер

[r5] Безработный

[r6] Нетрудоспособный

[r7] Ничего из перечисленного (опишите):

COMPLETE - testing

Your responses will NOT be saved.

You may now close this window or use the "Back" button to keep testing the survey.
